# Class E sortase SrtE and two SrtE-dependent cell wall-anchored hydrophobic proteins are involved in morphogenesis in *Actinoplanes missouriensis*: occurrence of exploratory growth beyond genus *Streptomyces*

**DOI:** 10.1128/mbio.03944-25

**Published:** 2026-05-18

**Authors:** Zhuwen Tan, Kazuki Nosho, Kyohei Umebayashi, Kenji Akamatsu, Reiichi Ariizumi, Takeaki Tezuka, Yasuo Ohnishi

**Affiliations:** 1 Department of Biotechnology, Graduate School of Agricultural and Life Sciences, The University of Tokyo 13143, Bunkyo-ku, Tokyo, Japan; 2 Collaborative Research Institute for Innovative Microbiology, The University of Tokyo 595461 , Bunkyo-ku, Tokyo, Japan; 3 CarbGeM Inc., Shibuya-ku, Tokyo, Japan; Friedrich-Schiller-Universitat, Jena, Germany

**Keywords:** *Actinoplanes missouriensis*, cell surface protein, exploration, sortase, sporangium formation

## Abstract

The actinomycete *Actinoplanes missouriensis* grows by extending branched hyphae and forms terminal sporangia containing a few hundred spores. A sortase-dependent spore surface protein, SspA, has recently been shown to be important for sporangium maturation in *A. missouriensis*. In this study, we functionally characterized all five sortase candidates in *A. missouriensis* and revealed that only a class E sortase (SrtE) is required for sporangium formation. The *srtE* null mutant strain scarcely produced sporangia, in contrast to the *sspA* null mutant, which produced fragile sporangia, indicating that there are other SrtE substrate proteins besides SspA. Unexpectedly, Δ*srtE* colonies displayed a significantly increased rate of surface area expansion on nutrient-rich solid media compared with wild-type colonies. The growing hyphae of the Δ*srtE* strain raised the pH of the surrounding environment, possibly by emitting basic volatile compounds. This growth mode shares several defining characteristics with the exploratory growth (or exploration) of *Streptomyces*. We identified two SrtE-dependent cell wall-anchored proteins (CwpA and CwpB) required for the suppression of latent exploration in the wild-type strain; colonies of single-gene mutants of *cwpA* and *cwpB* showed exploration. While the Δ*cwpA* strain formed normal sporangia, severe defects in the initiation of sporangium formation were observed in the Δ*cwp*B strain, indicating that CwpB, but not CwpA, is essential for sporangium formation. Based on these results, we propose that SrtE suppresses latent exploration and promotes the initiation of sporangium formation by making the cell surface hydrophobic through the attachment of the hydrophobic proteins CwpA and CwpB to the cell wall.

**IMPORTANCE:**

Exploration has recently been established as a new growth mode in the filamentous actinobacterial genus *Streptomyces*, where rapidly expanding mycelia lead to extensive surface colonization on nutrient-rich solid media. However, the phylogenetic prevalence and genetic basis of this unique growth mode remain unclear. Here, we report the discovery of exploration in *Actinoplanes missouriensis*, a filamentous actinomycete that forms terminal sporangia and zoospores. Through extensive genetic experiments, we demonstrated that a class E sortase suppresses latent exploration via covalent anchoring of two surface hydrophobic proteins to the cell wall in *A. missouriensis*. This study revealed that exploration capabilities are prevalent among filamentous actinomycetes beyond *Streptomyces* and that the hydrophobic nature of the cell surface appears to be crucial for suppressing latent exploration in *A. missouriensis*. The hydrophobic nature of the cell surface also appears to be important for initiating sporangium formation on nutrient-poor solid media in *A. missouriensis*.

## INTRODUCTION

Members of the genus *Streptomyces* are gram-positive actinobacteria that are renowned for their complex multicellular sporulating life cycle. Following spore germination, vegetative mycelium grows by hyphal tip extension and branching until reproductive growth is initiated, which involves raising aerial hyphae from vegetative mycelia and subsequently converting the aerial hyphae into spore chains (1). In addition to this developmental life cycle, a new growth mode, termed exploratory growth or exploration, has been described for *Streptomyces* bacteria, which is characterized by the rapid outgrowth of non-branching vegetative hyphae across a solid surface (2–6). The universally shared characteristics of exploration in *Streptomyces* bacteria are as follows: (i) a significantly increased rate of surface area expansion on solid medium, (ii) unique colony surface morphologies characterized by wrinkles, blisters, and buckles, and (iii) requirement for the function of the sigma factor SigE (7). SigE is a key regulator of the cell envelope stress response in *Streptomyces* species (8–10). Except for exploration on malt extract-yeast extract-maltose with added glycerol (MYMG) agar, the emission of trimethylamine, which raises the pH of the surrounding environment, is also a universally observed characteristic during exploration. This alkalinization induces exploration in separately located *Streptomyces* and reduces the bioavailability of iron, thereby limiting the growth of other nearby microbes (2, 11, 12). Concerning genetic control, exploration on MYMG agar was arrested in the *bldD* null and *rsiG* null mutant strains of *Streptomyces venezuelae*, possibly due to the inappropriately high activity of the response regulator WhiI, which is required for efficient sporulation septation, in both strains (7). The *bldD* and *rsiG* genes encode a global transcriptional regulator repressing the initiation of morphological development and a cognate anti-sigma factor of the sporulation-specific sigma factor WhiG, respectively (7). Recently, Zambri et al. reported that *S. venezuelae* uses both DivIVA-mediated polar growth and MreB1-mediated dispersed cell wall synthesis during exploratory growth on yeast extract-peptone (YP) agar (13).

Sortases are membrane-bound cysteine transpeptidases that catalyze the anchoring of surface proteins to the cell wall peptidoglycan. Generally, sortase substrate proteins possess a C-terminal sorting signal consisting of a pentapeptide LPXTG (X, any amino acid) motif and a transmembrane region followed by a stretch of basic residues. Sortases recognize and cleave the sorting signal (between the fourth T and fifth G of the pentapeptide) to covalently attach substrate proteins to a peptidoglycan precursor, lipid II, by forming a peptide bond between the substrate proteins and lipid II (14, 15). Sortases are classified into six groups (classes A–F) based on their primary sequence (16). Class A sortases play housekeeping roles, mostly in low-GC gram-positive bacteria (14, 16). Class B sortases are responsible for iron uptake by anchoring iron transporters to the cell wall of *Bacillus* and *Listeria* species (16). Class C sortases participate in pili assembly (14, 16, 17). A member of the class D sortases is involved in spore formation in *Bacillus anthracis* (16, 18). Class E sortases are found in actinobacteria and are involved in aerial hyphae formation in *Streptomyces* (16). The first example of class F sortases was reported in *Propionibacterium acnes*; however, their physiological roles remain unknown (16, 19).

*Actinoplanes missouriensis* is the model species of the genus *Actinoplanes*, members of which are characterized by a life cycle involving complex morphological development (20). *A. missouriensis* forms a branched substrate mycelium during vegetative growth and subsequently produces globose or subglobose terminal sporangia that grow from the substrate mycelium via short sporangiophores on humic acid-trace element (HAT) agar (21, 22). Each sporangium contains a few hundred spores, and the confined space between spores inside a sporangium is filled with an intrasporangial matrix called the sporangium matrix. In response to water exposure, sporangia open and release spores through a process called sporangium dehiscence (23, 24). After release from sporangia, spores disperse in aquatic environments as zoospores that swim using flagella and exhibit chemotactic responses (25–27). At a niche suitable for vegetative growth, zoospores stop swimming and begin germination (28). On HAT agar, small sporangium-like structures are formed after 2 or 3 days of cultivation at 30°C. Mature sporangia that can release spores under sporangium dehiscence-inducing conditions are formed after 5–7 days of cultivation.

Recently, we reported that deletion of *sspA*, which encodes a putative intrinsically disordered protein, causes the formation of fragile sporangia in *A. missouriensis* (29). The sporangia of the *sspA* null mutant (Δ*sspA*) strain were easily disrupted during sample preparation for scanning electron microscopy (SEM), and many naked spores were observed (29). Furthermore, when Δ*sspA* sporangia were suspended in a 50 mM NaCl solution, in which spores were not released from wild-type sporangia, many spores were detected in the solution, indicating that Δ*sspA* sporangia are very fragile and easily collapse and release spores without normal sporangium dehiscence processes (29). As SspA contains a C-terminal sorting signal and localizes to the surface of zoospores, sortase is likely to be involved in its surface anchoring (29). However, this sortase has not yet been identified. Therefore, in this study, we functionally characterized all five sortase candidates encoded in the *A. missouriensis* genome by gene disruption and revealed that a class E sortase, named SrtE, plays a crucial role in sporangium formation on HAT agar. No phenotypic changes were observed in the quadruple gene deletion mutant of the remaining four sortase candidate genes, all of which encoded class F sortases, indicating that the sortase used for SspA cell wall localization is SrtE. Interestingly, loss of SrtE induced a markedly different growth mode from that of the wild-type strain on nutrient-rich yeast extract-beef extract-NZ amine-maltose monohydrate (YBNM) agar. This growth mode shares several key characteristics with the exploratory growth of *Streptomyces* bacteria. Finally, we revealed that two proteins with a C-terminal sorting signal, named CwpA and CwpB, play crucial roles in the phenotypic changes induced by the loss of SrtE.

## RESULTS

### *A*. *missouriensis* contains one class E and four class F sortase candidates

In the *A. missouriensis* chromosome, we found five sortase gene candidates (*AMIS_500*, *AMIS_11000*, *AMIS_17370*, *AMIS_33280*, and *AMIS_36430*), which encode proteins of 792, 273, 184, 240, and 247 amino acids, respectively. A protein database search using InterPro ver. 106.0 (https://www.ebi.ac.uk/interpro/) revealed that AMIS_500 has a bacterial sortase class E domain (accession number IPR053465; residues 575–775) and that AMIS_11000, AMIS_17370, AMIS_33280, and AMIS_36430 harbor a sortase class F domain (IPR042001; residues 120–266, 40–182, 92–237, and 98–244, respectively). Hereafter, we named the class E sortase (AMIS_500) SrtE and four class F sortases (AMIS_11000, AMIS_17370, AMIS_33280, and AMIS_36430) SrtF1, SrtF2, SrtF3, and SrtF4, respectively. We constructed a phylogenetic tree using 26 sortases from various gram-positive bacteria (*Actinomyces oris*, *Staphylococcus aureus*, *Bacillus cereus*, *Corynebacterium diphtheriae*, *P. acnes*, and *Streptomyces avermitilis*) and all five sortases from *A. missouriensis*. Consistent with the database search, SrtE and SrtF1–4 were found in the same clades as class E and class F sortases, respectively, from other bacteria (Fig. S1). On the *A. missouriensis* chromosome, the five sortase genes are located at separate loci (Fig. S2A).

### SrtE is required for sporangium formation

**Fig 1:**
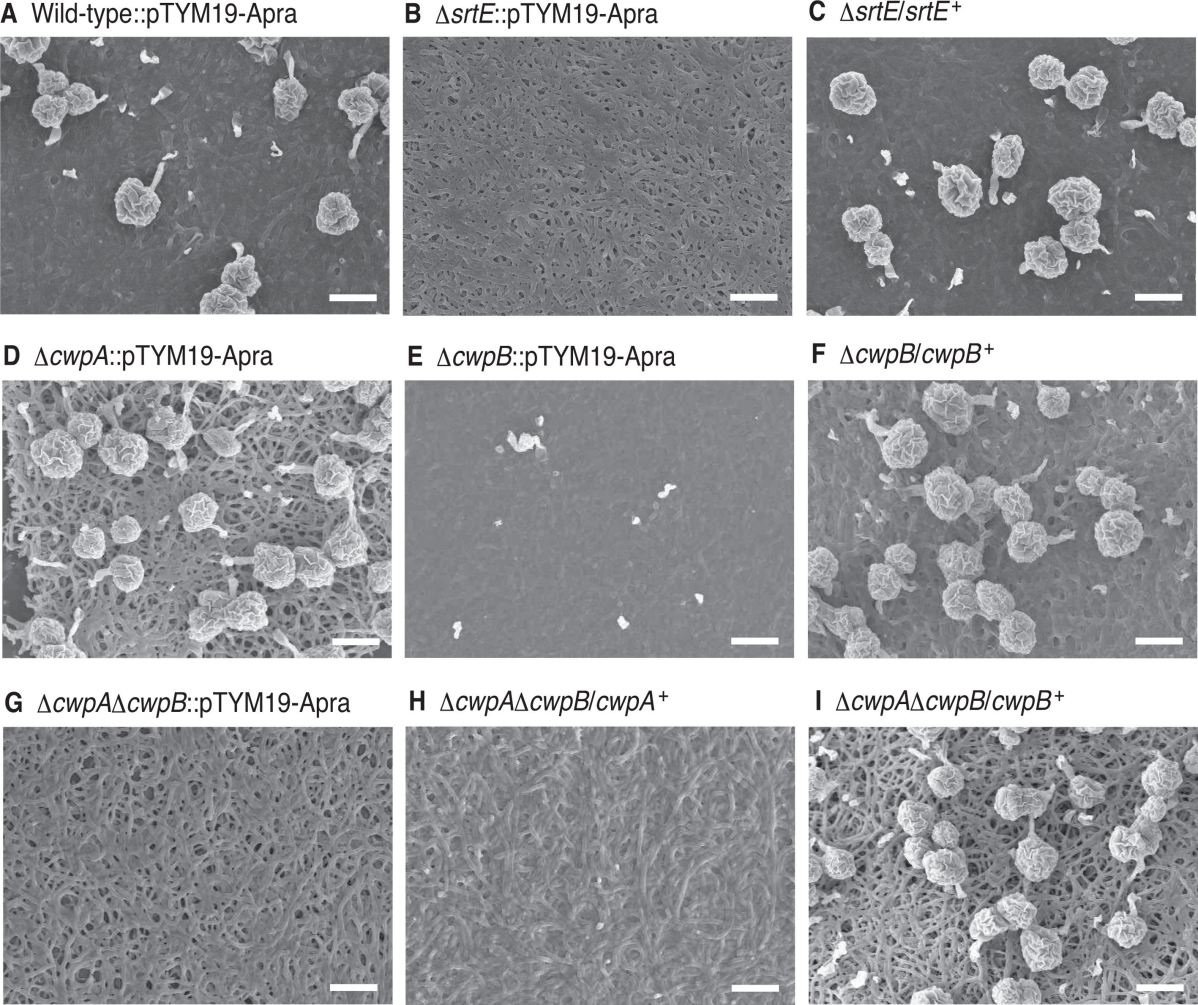
SEM analysis of mycelia and sporangia produced on HAT agar after 7 days of cultivation. (**A**) Wild-type strain harboring pTYM19-Apra. (**B**) Δ*srtE* strain harboring pTYM19-Apra. (**C**) Δ*srtE* strain harboring the *srtE* complementation plasmid. (**D**) Δ*cwpA* strain harboring pTYM19-Apra. (**E**) Δ*cwpB* strain harboring pTYM19-Apra. (**F**) Δ*cwpB* strain harboring the *cwpB* complementation plasmid. (**G**) Δ*cwpA*Δ*cwpB* strain harboring pTYM19-Apra. (**H**) Δ*cwpA*Δ*cwpB* strain harboring the *cwpA* complementation plasmid. (**I**) Δ*cwpA*Δ*cwpB* strain harboring the *cwpB* complementation plasmid. Bars, 5 µm.

**Fig 2:**
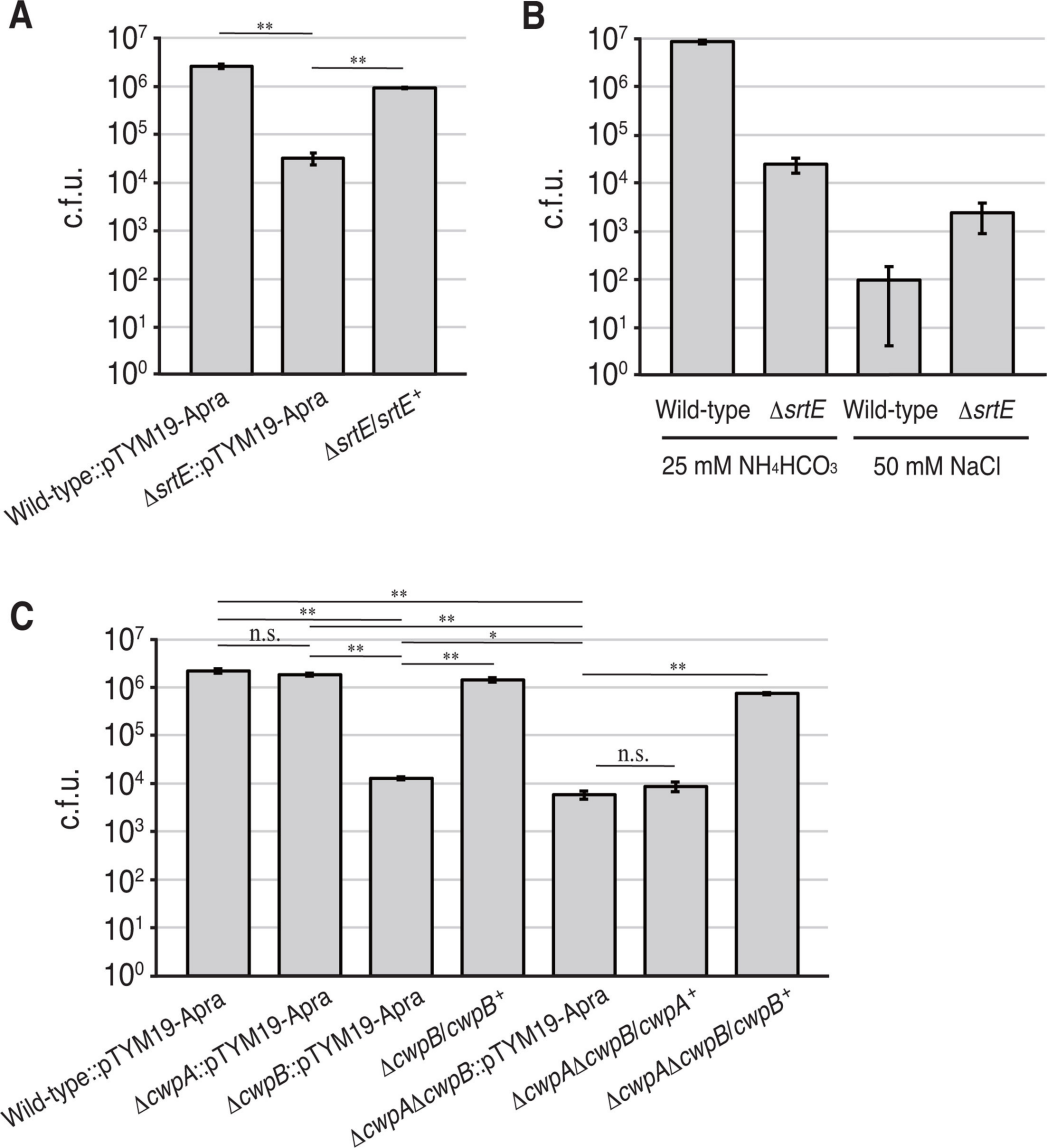
Number of spores released from sporangia. Each strain was cultivated on HAT agar at 30°C for 7 days. A 25 mM NH_4_HCO_3_ solution (**A–C**) or 50 mM NaCl solution (**B**) was poured onto a sporangium-forming agar plate, followed by incubation at room temperature for 1 h. The solution was retrieved from the agar surface and filtered through a 5-µm membrane filter to eliminate mycelia and sporangia. A portion of the zoospore suspension was cultivated on YBNM agar at 30°C for 2 days, and the number of colonies was counted to estimate the colony-forming unit (c.f.u.) value of the sample. The values represent the mean ± standard error of three biological replicates. (**A**) The wild-type and Δ*srtE* strains, both of which harbored pTYM19-Apra, and the Δ*srtE* strain harboring the *srtE* complementation plasmid. (**B**) The wild-type and Δ*srtE* strains. (**C**) The wild-type, Δ*cwpA*, Δ*cwpB*, and Δ*cwpA*Δ*cwpB* strains, all of which harbored pTYM19-Apra, the Δ*cwpB* strain harboring the *cwpB* complementation plasmid, and the Δ*cwpA*Δ*cwpB* strain harboring the *cwpA* or *cwpB* complementation plasmid. In panels **A** and **C**, differences were analyzed using Student’s *t*-test. * and ** mean *P* < 0.01 and *P* < 0.001, respectively. “n.s.” means no significant difference (*P* > 0.05).

To examine the *in vivo* functions, we generated single-gene mutants lacking one of the five putative sortase genes (Δ*srtE*, Δ*srtF1*, Δ*srtF2*, Δ*srtF3*, and Δ*srtF4* strains). Considering the possibility that the substrate proteins of four class F sortases overlap with each other, we generated six double mutants (Δ*srtF1*Δ*srtF2*, Δ*srtF1*Δ*srtF3*, Δ*srtF1*Δ*srtF4*, Δ*srtF2*Δ*srtF3*, Δ*srtF2*Δ*srtF4*, and Δ*srtF3*Δ*srtF4* strains), four triple mutants (Δ*srtF1*Δ*srtF2*Δ*srtF3*, Δ*srtF1*Δ*srtF2*Δ*srtF4*, Δ*srtF1*Δ*srtF3*Δ*srtF4*, and Δ*srtF2*Δ*srtF3*Δ*srtF4* strains), and one quadruple mutant (Δ*srtF1*Δ*srtF2*Δ*srtF3*Δ*srtF4* strain), which cover all combinations. We investigated sporangium formation and dehiscence in the mutant strains grown on HAT agar. SEM observations revealed that all mutants, except for the Δ*srtE* strain, formed normal sporangia, as observed in the wild-type strain (Fig. S3A through P). These mutant sporangia normally opened to release spores under sporangium dehiscence-inducing conditions (Fig. S4A through P and S5A through F). In contrast, sporangia were scarcely observed in the Δ*srtE* strain grown under the same conditions, indicating that the initiation of sporangium formation was severely inhibited in this mutant (Fig. 1A and B). Consistently, the number of spores released from Δ*srtE* sporangia was two orders of magnitude lower than that released from wild-type sporangia under sporangium dehiscence-inducing conditions (Fig. 2A). These phenotypic changes were restored by the introduction of *srtE* with its own promoter using a chromosome-integrating vector, pTYM19-Apra (30, 31), into the Δ*srtE* strain (Fig. 1C and 2A). These results indicate that SrtE plays an important role in the initiation of sporangium formation on HAT agar.

### SrtE is the only sortase responsible for SspA localization

As described in the Introduction, deletion of *sspA*, which encodes a sortase-dependent spore surface protein, causes the formation of fragile sporangia on HAT agar (29). As the Δ*srtF1*Δ*srtF2*Δ*srtF3*Δ*srtF4* strain produced normal sporangia, the four class F sortases were not responsible for SspA localization. Thus, we assumed that SrtE is the sortase responsible for SspA localization. However, it was difficult to observe fragile sporangia of the Δ*srtE* strain using SEM because it hardly produced sporangia; the number of sporangia was estimated to be approximately 1% of that of the wild-type strain from the colony-forming unit of released spores (Fig. 2A). To examine fragile sporangium formation, we utilized the phenomenon that normal sporangia do not release spores in 50 mM NaCl solution, while fragile sporangia easily collapse, and spores are observed in the solution. When incubated in 50 mM NaCl solution, Δ*srtE* sporangia released more than 10^3^ spores (per HAT plate), which was approximately 10% of the spores released under normal sporangium dehiscence-inducing conditions using 25 mM NH_4_CO_3_ solution, whereas wild-type sporangia released only 10^2^ spores (per HAT plate), which was approximately 0.001% of the spores released under normal sporangium dehiscence-inducing conditions (Fig. 2B). This result suggested that Δ*srtE* sporangia are fragile. Thus, we concluded that SrtE is responsible for SspA localization at the cell wall and that other SrtE substrate proteins are required to initiate sporangium formation.

### Lack of SrtE induces exploration

**Fig 3:**
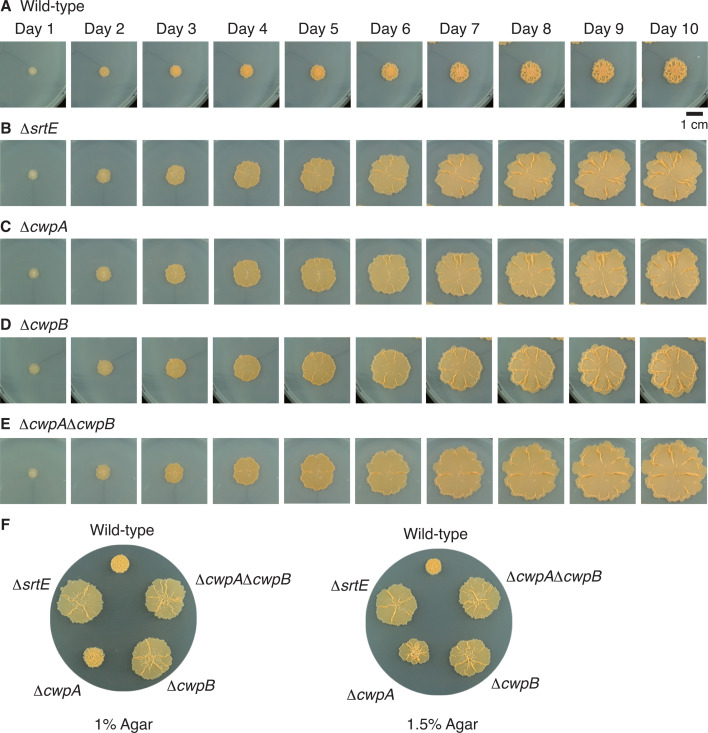
Mycelial growth on agar medium. (**A–E**) Colonies formed on agar medium were photographed at 24 h intervals. The wild-type (**A**), Δ*srtE* (**B**), Δ*cwpA* (**C**), Δ*cwpB* (**D**), and Δ*cwpA*Δ*cwpB* (**E**) strains were cultivated on YBNM solid medium containing 2.0% agar at 30°C for 10 days. (**F**) Colonies formed on solid medium containing different agar concentrations. The wild-type, Δ*srtE*, Δ*cwpA*, Δ*cwpB*, and Δ*cwpA*Δ*cwpB* strains were cultivated on YBNM solid medium containing 1.0% (left panel) or 1.5% (right panel) agar at 30°C for 5 days.

**Fig 4:**
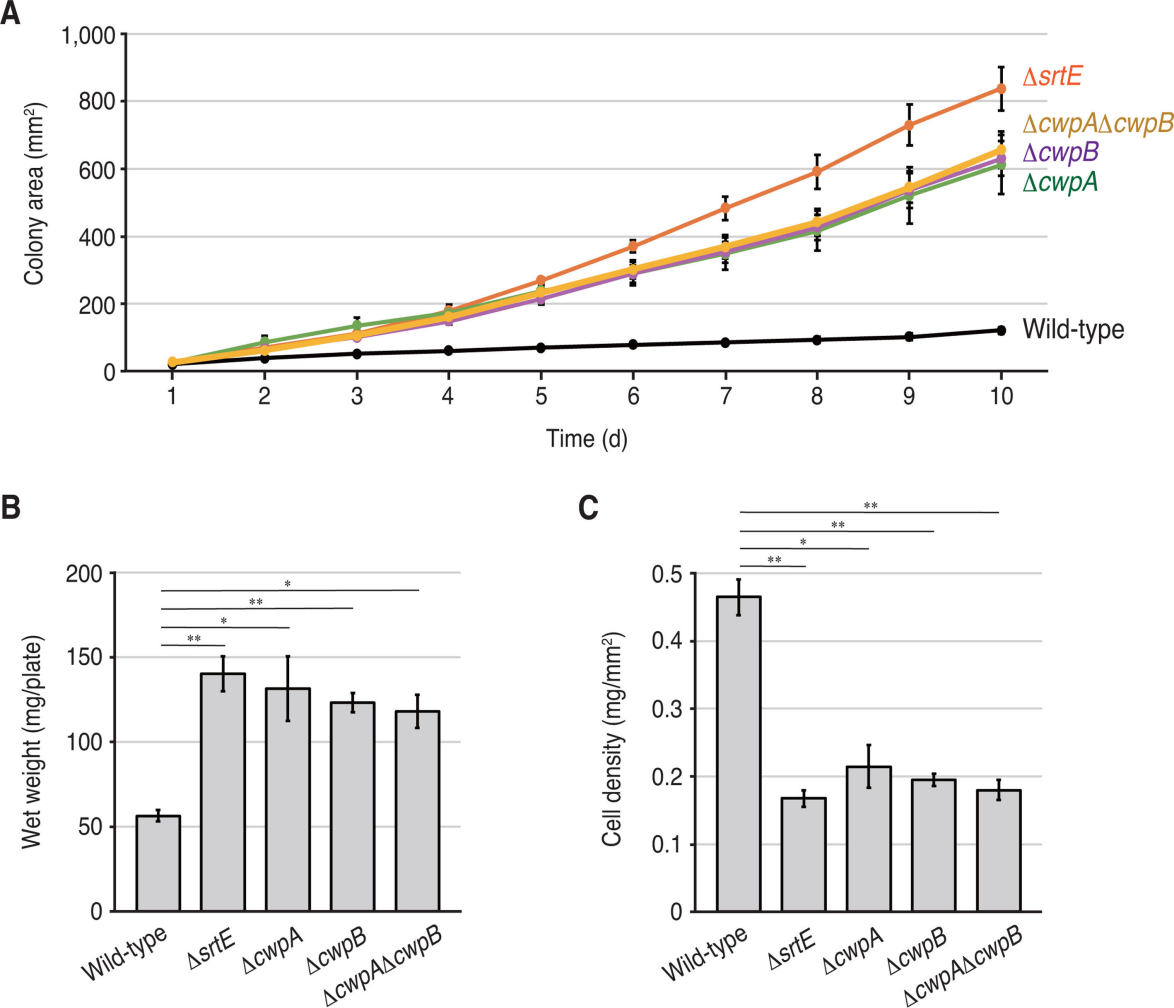
Colony expansion on agar medium. The wild-type, Δ*srtE*, Δ*cwpA*, Δ*cwpB*, and Δ*cwpA*Δ*cwpB* strains were cultivated on YBNM agar at 30°C for 10 days. (**A**) Quantification of the colony area measured at 24 h intervals. (**B**) Wet weights of mycelia harvested from the agar surface. Wet cell weights per plate were measured after 10 days of cultivation. (**C**) Cell densities of colonies were quantified based on colony area and wet cell weight. In panels **A**–**C**, the values represent the mean ± standard error of three biological replicates. In panels **B** and **C**, differences were analyzed using Student’s *t*-test. * and ** mean *P* < 0.01 and *P* < 0.001, respectively. No significant differences were observed among the Δ*srtE*, Δ*cwpA*, Δ*cwpB*, and Δ*cwpA*Δ*cwpB* strains (*P* > 0.05).

Unexpectedly, we found that the Δ*srtE* strain generated larger colonies than the wild-type strain on YBNM agar (Fig. 3A and B). Quantification of the colony area formed on YBNM agar demonstrated that the Δ*srtE* colonies were approximately 7-fold larger than the wild-type colonies after 10 days of cultivation at 30°C (Fig. 4A). The wet weight of Δ*srtE* mycelia collected from the agar plate after 10 days of cultivation was 2.5-fold higher than that of the wild-type strain (Fig. 4B). Thus, the cell density (i.e., wet weight of mycelia per colony area, which reflects colony thickness) of the wild-type strain was 2.8-fold higher than that of the Δ*srtE* strain, indicating that the wild-type strain formed a thicker colony than the mutant colony (Fig. 4C). These data suggest that Δ*srtE* mycelia expanded at the expense of cell density inside the colonized area. It should be noted that the mycelia of *S. venezuelae* during exploratory growth showed a similar ratio of increase in surface area and wet weight (7). Consistent with the macroscopic observation, long, straight, non-branching hyphae were observed by SEM at the edge of the Δ*srtE* colonies grown on YBNM agar (Fig. S6A and B). A gene complementation test confirmed that the introduction of *srtE* with its own promoter considerably reduced the size of colonies in the Δ*srtE* strain (Fig. S7). These results indicate that the vegetative hyphae of the Δ*srtE* strain rapidly grow and expand across solid medium surfaces, which is a defining characteristic of exploration. Notably, an increased rate of surface area expansion was not observed for the Δ*srtE* strain on HAT agar, possibly because vigorous mycelial growth did not occur on this nutrient-poor solid medium.

**Fig 5:**
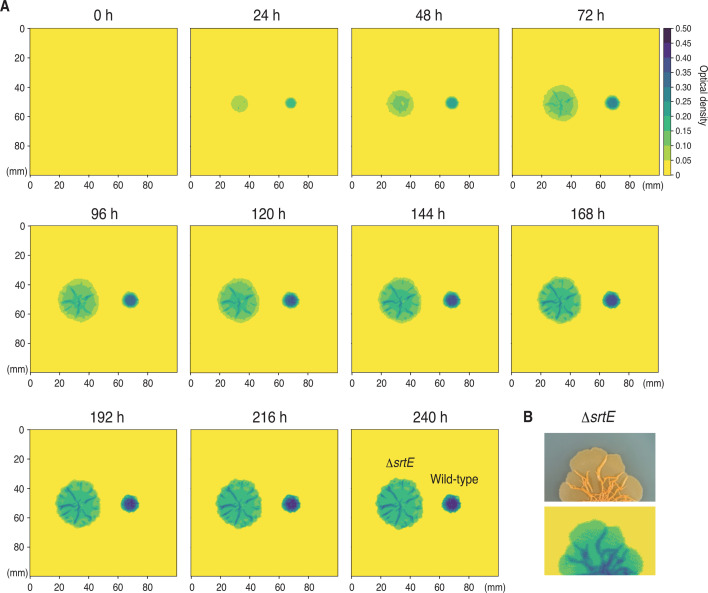
Optical density of colonies formed on the agar medium. (**A**) Heatmaps showing the optical density of colonies on YBNM agar. Suspensions of the wild-type (right side) and Δ*srtE* (left side) mycelia were inoculated and incubated at 30°C for 10 days. The optical density was measured with a resolution of 100 × 100 µm at intervals of 5 min, and the images at intervals of 24 h are shown. The data were visualized using heatmaps, and the color range is shown at the upper right of the images. (**B**) Colony morphology of the Δ*srtE* strain after 240 h of inoculation. Suspension of the Δ*srtE* mycelia was inoculated on YBNM agar and incubated at 30°C for 10 days. The upper and lower panels show the photograph and corresponding heatmap of the optical density, respectively.

To further characterize the significantly increased rate of surface area expansion in the Δ*srtE* strain, we analyzed growing colonies of the wild-type and Δ*srtE* strains on YBNM agar using time-lapse imaging. In this experiment, the optical density of the colonies on the solid medium was measured with a resolution of 100 × 100 µm at intervals of 5 min using a special apparatus, Bacterial Growth Monitor. The obtained data clearly showed that the Δ*srtE* strain rapidly expanded over a larger area than the wild-type strain at the expense of cell density inside the colonized area (Fig. 5A; Movie S1). They also showed that the Δ*srtE* colony developed a unique surface morphology with wrinkles and buckles (Fig. 5A and B), which is a universally shared characteristic of exploration in *Streptomyces*. In the case of *A. missouriensis*, however, the wild-type colony also developed a complex surface morphology with wrinkles and buckles (Fig. 3A), which is a somewhat different situation from that of *S. venezuelae* (7). The Δ*srtE* colony grown on YBNM agar expanded over a polystyrene barrier (Fig. S8A and B), as observed in *S. venezuelae* during exploration (2). Furthermore, the Δ*srtE* strain raised the pH of the surrounding environment during colony growth (Fig. S9A). This alkalinization seemed to be mediated by volatile compounds emitted from the Δ*srtE* colony because the physically separated solid medium was also alkalinized during the colony growth of the Δ*srtE* strain (Fig. S9B). Taken together, these observations clearly demonstrate that the lack of SrtE induced exploration in *A. missouriensis*. It should be noted that exploration was also triggered at the edge of the wild-type colony with an increasing pH of the surrounding solid medium after prolonged incubation (Fig. S9A; see Discussion).

### Hyphae of the Δ*srtE* strain cannot penetrate the agar plates

**Fig 6:**
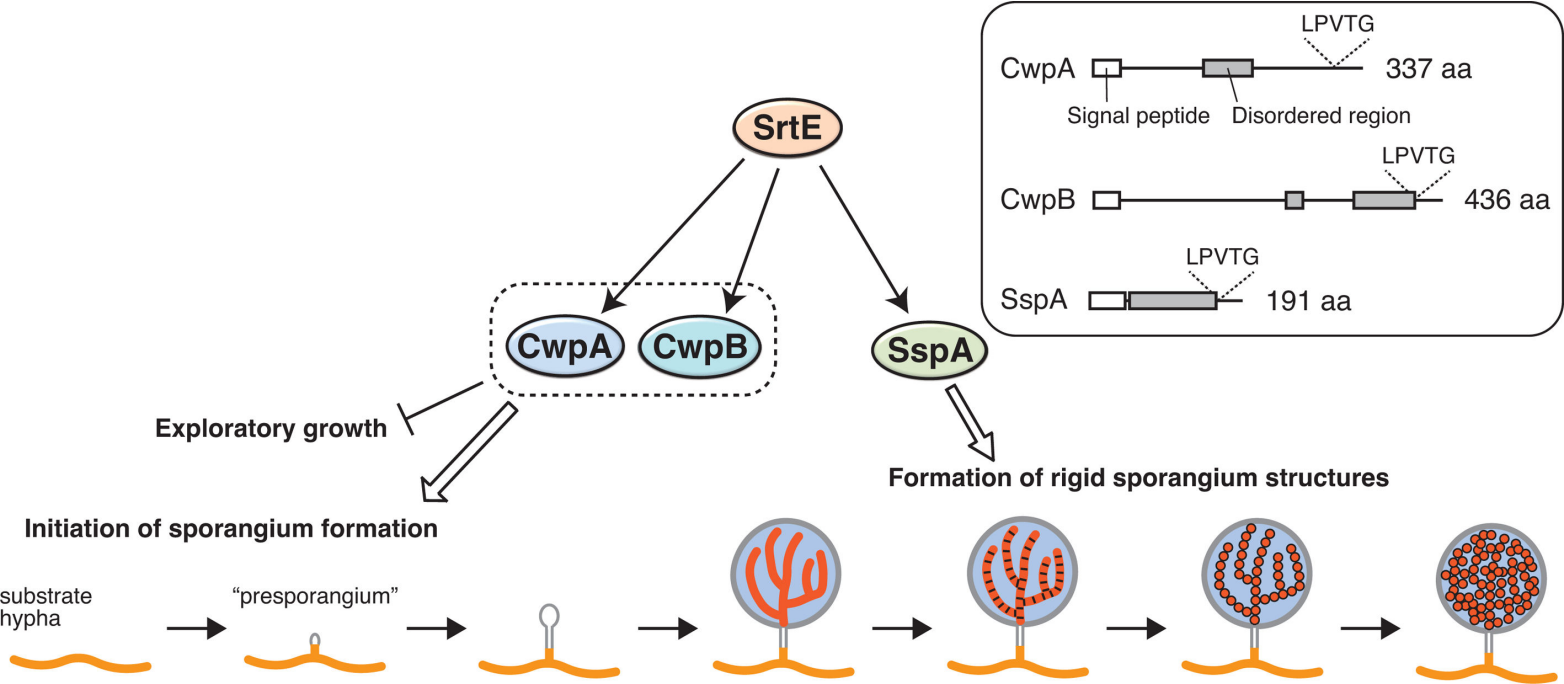
Regulatory model of morphological development by sortase and cell surface proteins in *A. missouriensis*. At the bottom of the figure, a proposed model of sporangium formation is illustrated. Domain organization and the SrtE-recognizing motif of CwpA, CwpB, and SspA are also illustrated in the upper right. SrtE sortase catalyzes the anchoring of the cell surface-exposed proteins CwpA, CwpB, and SspA to the cell wall. White arrows indicate the involvement of the gene products in the developmental stages shown in the diagram. A line with a bar indicates the repression of the biological phenomenon.

During observations of the Δ*srtE* colony on HAT and YBNM agar, we found that the attachment of Δ*srtE* mycelia to agar was much weaker than that of wild-type mycelia. For example, Δ*srtE* mycelia detached easily from the agar surface upon exposure to water (Fig. S10A and B). This result clearly indicates that the hyphae of the Δ*srtE* strain could not penetrate the agar layer. This “no-penetration” nature of mycelium seems to contribute to exploration. As described in the introduction, sortases attach their substrate proteins to the cell wall. This indicates that sortases alter certain characteristics of the cell surface. Therefore, we expect that the lack of StrE-dependent cell wall-anchored proteins changes the nature of the hyphal surface, resulting in the “no-penetration” phenotype and exploration. We further believe that the change in hyphal surface nature has negative effects on the onset of sporangium formation, in which a small round structure (presporangium; Fig. 6) is formed at the tip of a short hypha generated by branching.

### Two SrtE-dependent cell surface-exposed proteins, CwpA and CwpB, are required to suppress exploration

As described above, the lack of SrtE induced exploration on YBNM agar and inhibited the initiation of sporangium formation on HAT agar. In contrast, the lack of SspA, which is the substrate of SrtE, did not induce exploration on YBNM agar, although the Δ*sspA* strain formed fragile sporangia on HAT agar (29). Thus, we postulated that SrtE-dependent cell surface-exposed proteins other than SspA should suppress latent exploration and induce the initiation of sporangium formation in *A. missouriensis*. As described in the Introduction, sortase substrate proteins possess a pentapeptide motif, a transmembrane region, and a stretch of basic residues at their C-terminal region in this order. According to these criteria, we identified seven candidates for genes encoding sortase substrate proteins in the *A. missouriensis* genome: *AMIS_3380* (*cwpC*; cell wall-anchored protein C), *AMIS_17240* (*cwpD*), *AMIS_24730* (*cwpE*), *AMIS_28200* (*cwpF*), *AMIS_57330* (*cwpG*), *AMIS_70730* (*cwpA*), and *AMIS_70740* (*cwpB*), which encode proteins of 460, 509, 762, 512, 219, 337, and 436 amino acids, respectively, in addition to *sspA* (Fig. S11A through H; see Fig. 6 for CwpA and CwpB) (29). On the *A. missouriensis* chromosome, five sortase genes (*srtE* and *srtF1–4*), seven sortase substrate candidate genes (*cwpA–G*), and *sspA* are located on separate loci, with the exception that *cwpA* and *cwpB* appear to form an operon in this order (Fig. S2A and B). It should be noted that *AMIS_11010* and *AMIS_36440*, which are located on the adjacent loci to *srtF1* and *srtF4*, respectively, encode functionally unknown proteins that possess a signal peptide for the general secretion pathway at their N-terminal region, a transmembrane region, and a stretch of basic residues at their C-terminal region (Fig. S11I and J). Because the sortase-recognizing pentapeptide motifs were not found in the gene products of *AMIS_11010* and *AMIS_36440*, these genes were not considered sortase substrate candidate genes and were not analyzed further in the present study. However, considering the genetic context, *AMIS_11010* and *AMIS_36440* may encode substrates of SrtF1 and SrtF4, respectively.

A database search using InterPro predicted that the extracellular domains of CwpA–F contain disordered regions (residues 143–203 for CwpA; residues 238–258 and 324–399 for CwpB; residues 101–143, 263–322, and 384–417 for CwpC; residues 367–451 for CwpD; residues 267–292 and 662–724 for CwpE; residues 196–243 and 429–472 for CwpF) (Fig. S11 and S12; see Fig. 6 for CwpA and CwpB). CwpF and CwpG harbor an ABC transporter-like domain (IPR022395; residues 200–508) and a neocarzinostatin-like domain (IPR002186; residues 31–131), respectively (Fig. S11 and S12). The PASTA 2.0 server (http://old.protein.bio.unipd.it/pasta2/) predicted that CwpA and CwpB contain one and four amyloid-like regions (residues 261–269 for CwpA and residues 77–87, 201–208, 224–228, and 310–329 for CwpB), respectively (32). We expected that these candidates would include genes responsible for the suppression of exploration and initiation of sporangium formation. We then generated single-gene mutants lacking one of the seven genes (Δ*cwpA*, Δ*cwpB*, Δ*cwpC*, Δ*cwpD*, Δ*cwpE*, Δ*cwpF*, and Δ*cwpG* strains) and a double mutant of *cwpA* and *cwpB* (Δ*cwpA*Δ*cwpB* strain), considering the phenotypic changes observed in the Δ*cwpA* and Δ*cwpB* strains (see below). No differences were observed in mycelial growth on YBNM agar among the wild-type, Δ*cwpC*, Δ*cwpD*, Δ*cwpE*, Δ*cwpF*, and Δ*cwpG* strains. In contrast, colonies of the Δ*cwpA*, Δ*cwpB*, and Δ*cwpA*Δ*cwpB* strains showed a significantly increased rate of surface area expansion on YBNM agar, which was similar to that of the Δ*srtE* strain (Fig. 3B through E; Fig. 4A). Based on the wet weight of mycelia, the cell density of the wild-type strain was 2.2-fold, 2.4-fold, and 2.6-fold higher than that of the Δ*cwpA*, Δ*cwpB*, and Δ*cwpA*Δ*cwpB* strains, respectively (Fig. 4B and C). Consistent with this result, long, straight, non-branching hyphae were observed at the edges of Δ*cwpA*, Δ*cwpB*, and Δ*cwpA*Δ*cwpB* colonies (Fig. S6C through E). Similar to Δ*srtE* mycelia, Δ*cwpA*Δ*cwpB* mycelia detached easily from the agar surface upon exposure to water (Fig. S10B). Notably, the rate of surface area expansion in the Δ*cwpA* strain was low on YBNM solid medium containing a lower amount (1.0% or 1.5%; wt/vol) of agar, compared with that on YBNM solid medium containing 2.0% (wt/vol) agar used for all other experiments in this study (Fig. 3F). This phenomenon was not observed in the Δ*srtE*, Δ*cwpB*, and Δ*cwpA*Δ*cwpB* strains, indicating that CwpA has a smaller effect on the suppression of exploration than CwpB. Because hyphal penetration into the agar seems to become easier at lower agar concentrations, it is convincing that decreasing agar concentration generates more difficult conditions for exploration. The Δ*cwpA*, Δ*cwpB*, and Δ*cwpA*Δ*cwpB* strains raised the pH of the surrounding areas during colony growth on YBNM agar (Fig. S9A). These observations strongly support the notion that the lack of CwpA and/or CwpB is responsible for the exploration observed in the Δ*srtE* strain, indicating that the attachment of CwpA and CwpB to the cell wall is catalyzed by SrtE.

### Comparison of CwpA and CwpB with long chaplins from *Streptomyces*

The amino acid sequence identity and similarity between the mature polypeptides of CwpA and CwpB are 19% and 29%, respectively. They share hydrophobic properties with long chaplins (ChpA–C) from *Streptomyces coelicolor* A3(2) (Fig. S13). Long chaplins (ChpA–C; Fig. S12) are sortase-dependent cell wall-anchored proteins that, together with short chaplins (ChpD–H), enable aerial hyphae to extend into the air in *Streptomyces* (1, 33). The long chaplins harbor two chaplin domains, each of which is composed of multiple β-sheets. The chaplin domain is highly hydrophobic and contains three GN motifs spaced 12–13 residues apart and two cysteine residues, with the exception that the two cysteine residues are not conserved in the C-terminal chaplin domain of ChpB (34) (Fig. S14C through E). The GN residues are located at the turns connecting individual β-sheets. An amino acid sequence alignment of CwpA, CwpB, and long chaplins (ChpA–C) revealed that CwpB had two conserved cysteine residues that corresponded to those in the N-terminal chaplin domains of ChpA–C (Fig. S15). However, no GN motifs were conserved around them. The alignment also revealed that CwpB had a GN motif that may correspond to the third GN motif in the C-terminal chaplin domains of ChpA–C (Fig. S15). However, the other two GN motifs and Cys residues were not conserved. Meanwhile, the mature CwpA possesses four cysteine residues (Cys-44, Cys-146, Cys-213, and Cys-292), although none of these are conserved in CwpB and ChpA–C (Fig. S15). CwpA also contained no GN motifs. Based on these results, we concluded that CwpA and CwpB have no chaplin domains. However, it should be noted that the AlphaFold-predicted structures of the mature parts of CwpA, CwpB, and long chaplins (ChpA–C) have similar characteristics: large disordered region(s) and a few clusters of β-sheets (Fig. S14), although the amino acid sequence identities and similarities among these surface proteins are low (10%–19% and 13%–29%, respectively; Fig. S15). When we compared CwpA or CwpB with long chaplins (ChpA–C), the identities and similarities were 10%–15% and 13%–20%, respectively (Fig. S15). Taken together, we believe that CwpA and CwpB make the hyphal surface hydrophobic, similar to long chaplins. Notably, no homologs with significant amino acid sequence identities to mature CwpA or CwpB are encoded in the *A. missouriensis* genome, in contrast to the chaplin system, which is composed of multiple chaplin proteins, including short chaplins. The hydrophobic nature caused by the hyphal surface location of CwpA and CwpB may suppress latent exploration in the wild-type strain.

### Conservation of CwpA and CwpB among *Actinoplanes* and related genera

We performed a BLAST search of the NCBI genome database (https://blast.ncbi.nlm.nih.gov/Blast.cgi) and found that CwpA and CwpB homologs are conserved in at least 32 (64%) and 33 (66%), respectively, out of 50 *Actinoplanes* species whose genome sequences and gene annotations have been registered in the database. The amino acid sequence identities and similarities between CwpA and any of CwpA homologs are 27%–43% and 39%–57%, respectively, and those between CwpB and any of CwpB homologs are 21%–66% and 31%–78%, respectively. Furthermore, proteins showing high similarities to CwpA and CwpB are also conserved in other actinomycete species, including *Pseudosporangium ferrugineum*, *Nucisporomicrobium flavum*, and *Winogradskya consettensis*. Meanwhile, CwpA homologs, but not CwpB homologs, are conserved in actinomycete species, including *Couchiplanes caeruleus*, *Krasilnikovia cinnamomea*, and *Paractinoplanes lichenicola. N. flavum* forms irregular pseudosporangia consisting of non-motile spores (35). *C. caeruleus* forms flagellated arthrospores by fragmentation of aerial hyphae (36). *K. cinnamomea* forms pseudosporangia on short sporangiophores above the surface of substrate mycelium (37). Members of the genera *Winogradskya* and *Paractinoplanes* are closely related to *Actinoplanes* (38). These homologs demonstrate that CwpA and CwpB homologs are evolutionarily conserved among the genus *Actinoplanes* and related actinomycetes. Meanwhile, CwpA or CwpB homologs are not conserved in members of the genus *Streptomyces* (less than 20% identity and 28% similarity).

### CwpA and CwpB are also involved in the initiation of sporangium formation

We analyzed the morphological development of the mutant strains. No differences were observed in sporangium formation and dehiscence among the wild-type, Δ*cwpC*, Δ*cwpD*, Δ*cwpE*, Δ*cwpF*, and Δ*cwpG* strains (Fig. S3A and Q through U, S4A and Q through U, and S5G and H). Unexpectedly, the Δ*cwpA* strain grown on HAT agar formed normal sporangia that opened and released spores similar to the wild-type sporangia (Fig. 1D; Fig. S4V; Fig. 2C). In contrast, severe defects in sporangium formation were observed in the Δ*cwpB* and Δ*cwpA*Δ*cwpB* strains; only a small number of distorted sporangium-like structures were formed in the Δ*cwpB* strain, and almost no sporangia were observed in the Δ*cwpA*Δ*cwpB* strain (Fig. 1E and G). Consistently, the number of spores released from Δ*cwpB* and Δ*cwpA*Δ*cwpB* sporangia was two orders of magnitude lower than that released from wild-type sporangia under sporangium dehiscence-inducing conditions (Fig. 2C). Notably, the number of spores released from Δ*cwpA*Δ*cwpB* sporangia was slightly lower than that released from Δ*cwpB* sporangia, indicating a minor but significant role of CwpA in sporangium formation and/or dehiscence (Fig. 2C). In the gene complementation test, *cwpB* was introduced into the Δ*cwpB* strain, and *cwpA* and *cwpB* were individually introduced into the Δ*cwpA*Δ*cwpB* strain using pTYM19-Apra. We found that normal sporangium formation and dehiscence were restored by the introduction of *cwpB* into the Δ*cwpB* and Δ*cwpA*Δ*cwpB* strains (Fig. 1F and I and 2C). The introduction of *cwpA* into the Δ*cwpA*Δ*cwpB* strain had almost no effect on sporangium formation and dehiscence (Fig. 1H and 2C). These results demonstrate that CwpB plays a crucial role in sporangium formation. However, considering the phenotypic changes between the Δ*cwpB* and Δ*cwpA*Δ*cwpB* strains, we concluded that CwpA is also involved in sporangium formation, although its contribution appears to be much smaller than that of CwpB.

## DISCUSSION

In this study, we demonstrated that the lack of SrtE induces exploration in *A. missouriensis*. To the best of our knowledge, this is the first report describing the relationship between exploration and sortase. We also identified two cell surface-localized hydrophobic proteins, CwpA and CwpB, as SrtE substrates that suppress exploration in the wild-type strain, although the molecular basis for exploration in *A. missouriensis* mutant strains requires further investigation. We consider the discovery of exploration capabilities in *A. missouriensis* significant because it raises the possibility that exploration is prevalent across diverse filamentous actinomycetes beyond *Streptomyces*. In this regard, it is noteworthy that prolonged incubation (e.g., more than 10 days) of the wild-type *A. missouriensis* strain on YBNM solid medium sometimes triggered exploration at the edge of the colonies, showing that exploration is not restricted to certain mutant strains (Fig. S16; see also Fig. S9A and S10B). In the developmental life cycle of *A. missouriensis*, sporangiospores function as a genetic repository, and their movement to new environments is mediated by the dispersal of zoospores into aquatic environments. Meanwhile, exploration allows growing mycelium to colonize new areas far from the site of germination under nutrient-rich solid conditions, where sporangium formation does not occur. Therefore, both zoospore formation and exploration are likely to be important survival strategies for *A. missouriensis*.

Previous work on *S. venezuelae* reported that exploring colonies produce and release trimethylamine to promote exploratory behavior in other streptomycetes and inhibit the growth of other microbes through environmental alkalinization (2, 11). In this study, we observed similar alkalinization in Δ*srtE*, Δ*cwpA*, Δ*cwpB*, and Δ*cwpA*Δ*cwpB* strains via the emission of alkaline compounds (Fig. S9). We assume that the volatile alkaline compound is trimethylamine; however, its identification awaits further experiments. Because wild-type colonies near the Δ*srtE* and Δ*cwpA*Δ*cwpB* strains grew normally without any change in colony morphology (Fig. 3F), the medium alkalinization induced by the volatile compound seems not to induce the exploration of the wild-type strain. Previous studies have also revealed that two siderophores, desferrioxamine and foroxymithine, make important contributions to exploration in *S. venezuelae* by sequestration of iron (7, 11, 12). Whether siderophores play a crucial role in the exploration of *A. missouriensis* requires further investigation.

While CwpA and CwpB function to suppress exploration in *A. missouriensis*, the sigma factor SigE is required for exploration in *S. venezuelae*, which upregulates a large regulon, including many cell envelope-associated enzyme genes (7, 10, 39). Key proteins under the control of SigE in *Streptomyces* include penicillin-binding proteins, L,D-transpeptidases, a LytR-CpsA-Psr-family protein involved in wall teichoic acid deposition, and a protein predicted to add lysyl groups to phosphatidylglycerol to neutralize the membrane charge (10). These findings suggest that cell surface properties are critical factors in exploration. Modulation of cell surface properties via the attachment of surface-exposed proteins appears to be an effective approach for the transition between exploration and conventional mycelial growth. In *S. coelicolor* A3(2), SigE is encoded in an operon along with *cseA*, *cseB*, and *cseC*, which encode a lipoprotein, response regulator, and sensor kinase, respectively (9). Transcription of *sigE* is dependent on the CseB/CseC two-component system and is induced by cell wall damage (8, 9, 40). On the *A. missouriensis* chromosome, 11 genes (*AMIS_1950*, *AMIS_17080*, *AMIS_18740*, *AMIS_22170*, *AMIS_23110*, *AMIS_24970*, *AMIS_26060*, *AMIS_29790*, *AMIS_34010*, *AMIS_57590*, and *AMIS_81010*) are predicted to encode SigE-family sigma factors, which share 25%–36% amino acid sequence identities with SigE in *S. coelicolor* A3(2). However, no gene clusters encoding a lipoprotein, response regulator, and sensor kinase were found in the loci adjacent to these sigma factor genes, suggesting that the CseB/CseC–SigE system does not exist in *A. missouriensis*.

Our genetic analysis revealed that the sortase SrtE is widely involved in the morphogenesis of *A. missouriensis* via the functions of its substrate proteins, SspA, CwpA, and CwpB (Fig. 6). This finding is significant because the functional analysis of class E sortase has been limited in *Streptomyces*. SrtE seems to recognize and cleave the LPVTG pentapeptide motif because this sequence is conserved in SspA, CwpA, and CwpB, whereas none of CwpC–G share this sequence (Fig. S11). Previously, we performed an RNA sequencing analysis at various time points during the life cycle of *A. missouriensis* (23). According to this analysis, the transcript levels of *cwp*A and *cwpB* were upregulated during vegetative growth and gradually downregulated during sporangium formation (Fig. S17). The transcript level of *sspA* was highly upregulated at the early stages of sporangium formation (Fig. S17) (29). These transcriptional profiles of *cwpA*, *cwpB*, and *sspA* closely correlated with the physiological functions of the gene products (Fig. 6). CwpA and CwpB repress exploration during vegetative growth and promote the initiation of sporangium formation, whereas SspA is involved in spore maturation inside sporangia (29). In contrast, *srtE* transcription was maintained at basal levels throughout sporangium formation and dehiscence (Fig. S17). Thus, transcriptional regulation of *cwpA*, *cwpB*, and *sspA* is likely to be one of the mechanisms that control the amount of surface-exposed CwpA, CwpB, and SspA proteins. Regulation at the post-translational level, including anchoring to the cell wall peptidoglycan catalyzed by SrtE, may also be involved in the control of the amount of these surface-displayed proteins.

Considering that *cwpA* and *cwpB* consist of an operon, it is reasonable that CwpA and CwpB have related functions, although the amino acid sequence identity of their mature polypeptides is low (19%). AlphaFold-Multimer ColabFold version (https://colab.research.google.com/github/sokrypton/ColabFold/blob/main/AlphaFold2.ipynb) proposed the possibility that CwpA and CwpB form a heterodimer complex through the interaction between the N-terminal, non-disordered regions of both proteins (Fig. S18). However, we speculate that at least the CwpB protein exerts its function by itself because the Δ*cwpA* strain was not deficient in sporangium formation, indicating that the presence of CwpB is sufficient for the onset of sporangium formation. Furthermore, the Δ*cwpA* strain did not show exploration on YBNM solid medium containing 1.0% agar, indicating that the presence of CwpB is sufficient for the suppression of exploration under this condition. Taken together, these findings indicate that CwpB has a greater impact on the morphogenesis of *A. missouriensis* than CwpA. Structural and biochemical analyses of CwpA and CwpB, as well as a more detailed characterization of the Δ*cwpA*Δ*cwpB* strain, are promising subjects for future research to obtain further insights into the molecular basis of exploration.

In conclusion, this study revealed the significant role of sortase SrtE in the morphogenesis of *A. missouriensis* (Fig. 6). Three SrtE-dependent cell wall-anchored proteins (SspA, CwpA, and CwpB) play important roles as structural proteins that directly affect the morphogenesis. CwpA and CwpB appear to make the hyphal surface hydrophobic, which suppresses exploration on nutrient-rich solid media and induces sporangium formation on nutrient-poor solid media. The discovery of exploration in *A. missouriensis* strongly indicates that exploration is widely distributed among diverse filamentous actinomycetes, beyond *Streptomyces*. Thus, this study provides new insights into the exploration, characteristic growth mode of filamentous actinomycetes, and morphological development for sporangium formation in *A. missouriensis*.

## MATERIALS AND METHODS

### General methods

Bacterial strains, plasmid vectors, and media used in this study have been described previously (31, 41, 42). Primers used in this study are listed in Table S1. *A. missouriensis* cells were prepared as previously described (43). SEM was performed using an S-4800 scanning electron microscope (Hitachi, Tokyo, Japan), as described previously (44). Phase-contrast microscopic observations of sporangia and zoospores were performed using a BH-2 microscope (Olympus, Tokyo, Japan), as described previously (45). Free zoospores were quantified as previously described (46).

### Identification of gene candidates encoding sortase substrate proteins

Genes predicted to encode sortase substrates were identified from the *A. missouriensis* proteome sequence according to the following criteria: (i) the gene product possesses a putative sortase-recognizing pentapeptide motif (LPXTG, NPXTG, SPXTG, PPXTG, LPXTA, or LAXTG; X, any amino acid) (17) and (ii) a transmembrane region and at least two consecutive basic residues (Arg, Lys, or His) are present in this order downstream from the pentapeptide motif at the C-terminal end.

### Construction of gene deletion mutants

In-frame markerless deletion mutant strains were constructed by replacing most of the coding sequences with the *Xba*I or *Pst*I recognition sequences, as follows. The upstream and downstream regions of target genes were amplified by PCR. Each amplified fragment was digested with appropriate restriction enzymes and cloned into pUC19. The generated plasmids were sequenced to confirm the absence of errors. The two cloned fragments were digested with appropriate restriction enzymes and cloned together into pK19mobsacB (47), whose kanamycin resistance gene had been replaced with the apramycin resistance gene *aac(3)IV*. The generated plasmids were introduced into *A. missouriensis* by conjugation as described previously (48). Apramycin-resistant colonies generated by single-crossover recombination were isolated. One of them was cultivated with shaking in peptone-yeast extract-magnesium (PYM) liquid broth at 30°C for 36–48 h, and the mycelia suspended in 0.75% NaCl solution were spread onto Czapek-Dox broth (BD, NJ, USA) agar medium containing extra sucrose (final concentration of 5%). After incubation at 30°C for 5 days, sucrose-resistant colonies were inoculated onto YBNM agar medium with or without apramycin to confirm their sensitivity to apramycin. Apramycin-sensitive and sucrose-resistant colonies generated by the second single-crossover recombination were isolated as candidates for gene deletion mutants. Deletion of the target genes was confirmed by PCR. The Δ*srtF1*Δ*srtF2*, Δ*srtF1*Δ*srtF3*, and Δ*srtF1*Δ*srtF4* strains were constructed by deleting *srtF2*, *srtF3*, and *srtF4*, respectively, using the Δ*srtF1* strain as the parental strain. The Δ*srtF2*Δ*srtF3* and Δ*srtF2*Δ*srtF4* strains were constructed by deleting *srtF3* and *srtF4*, respectively, using the Δ*srtF2* strain as the parental strain. The Δ*srtF3*Δ*srtF4* strain was constructed by deleting *srtF4* using the Δ*srtF3* strain as the parental strain. The Δ*srtF1*Δ*srtF2*Δ*srtF3* and Δ*srtF1*Δ*srtF2*Δ*srtF4* strains were constructed by deleting *srtF3* and *srtF4*, respectively, using the Δ*srtF1*Δ*srtF2* strain as the parental strain. The Δ*srtF1*Δ*srtF3*Δ*srtF4* strain was constructed by deleting *srtF4* using the Δ*srtF1*Δ*srtF3* strain as the parental strain. The Δ*srtF2*Δ*srtF3*Δ*srtF4* strain was constructed by deleting *srtF4* using the Δ*srtF2*Δ*srtF3* strain as the parental strain. The Δ*srtF1*Δ*srtF2*Δ*srtF3*Δ*srtF4* strain was constructed by deleting *srtF4* using the Δ*srtF1*Δ*srtF2*Δ*srtF3* strain as the parental strain.

### Construction of strains for gene complementation testing

The 3.0 and 1.3 kbp DNA fragments containing the promoter and coding sequences of *srtE* and *cwpA*, respectively, were amplified by PCR. For *cwpB*, DNA fragments containing the promoter sequence of *cwpA* and the coding sequence of *cwpB* were amplified by PCR, which were then connected to each other by overlap extension PCR. The amplified fragments were digested with *Eco*RI and *Hin*dIII, and cloned into pTYM19-Apra (30, 43) digested with the same restriction enzymes. The generated plasmids were sequenced to confirm the absence of PCR-derived errors. These plasmids were introduced into the Δ*srtE*, Δ*cwpA*, Δ*cwpB*, or Δ*cwpA*Δ*cwpB* strains by conjugation, as described previously (49). An empty vector, pTYM19-Apra, was also introduced into the wild-type and mutant strains. Apramycin-resistant colonies were obtained.

### Quantification of the area and optical density of colonies formed on the agar medium

The wild-type, Δ*srtE*, Δ*cwpA*, Δ*cwpB*, and Δ*cwpA*Δ*cwpB* strains were cultivated with shaking in PYM liquid broth at 30°C for 48 h, and the mycelia suspended in 0.75% NaCl solution (100 µL each) were inoculated onto YBNM agar medium. After the suspensions were desiccated, the inoculated agar was incubated at 30°C for 10 days and photographed at 24 h intervals. To quantify the colony areas, the growing colonies of each strain in the photographs were analyzed using ImageJ (50). To determine the optical density, suspensions of wild-type and Δ*srtE* mycelia were inoculated onto YBNM agar medium and cultivated at 30°C for 10 days. The optical density of the growing colonies was quantified using a Bacterial Growth Monitor (CarbGeM Inc., Tokyo, Japan). The Bacterial Growth Monitor measures the light transmission (wavelength: 468, 520, and 630 nm) of an agar plate with growing bacterial colonies using a device equipped with a two-dimensional thin-film transistor-based sensor with a resolution of 100 × 100 µm at 5 min intervals. Clusters of cells block light transmission, and the degree of this block in each pixel is defined as the optical density using the same calculation formula used for liquid culture. In this experiment, we analyzed the data obtained using light at 630 nm and produced a movie using heatmaps of every 4 h of data.

### Analysis of the pH of agar medium

To monitor the pH of agar medium during growth of the wild-type and mutant strains, suspensions of wild-type, Δ*srtE*, Δ*cwpA*, Δ*cwpB*, and Δ*cwpA*Δ*cwpB* mycelia were inoculated onto YBNM agar medium containing 83 mg/L bromothymol blue or 25 mg/L phenol red and cultivated at 30°C for 2 days. While colony growth was severely inhibited in all strains on the YBNM agar medium containing 83 mg/L bromothymol blue, colony growth at a similar rate to that on the YBNM agar medium without any pH indicator dye was observed on the YBNM agar medium containing 25 mg/L phenol red. Thus, suspensions of wild-type, Δ*srtE*, Δ*cwpA*, Δ*cwpB*, and Δ*cwpA*Δ*cwpB* mycelia (100 µL each) were inoculated onto YBNM agar medium containing 25 mg/L phenol red and cultivated at 30°C for 25 days. The inoculated agar plates were photographed on days 1, 5, 10, 15, 20, and 25 of the experiment. For the analysis of the pH of the physically separated agar medium, suspensions of wild-type and Δ*srtE* mycelia were inoculated onto a compartment of the plate with four compartments separated by polystyrene barriers. The inoculated agar plates were incubated at 30°C for 20 days and photographed on days 12, 16, and 20.

## SUPPLEMENTAL MATERIAL

File S1 mbio.03944-25-s0001.pdf Supplemental material title page; Figures S1 to S5.

File S2 mbio.03944-25-s0002.pdf Figures S6 to S8.

File S3 mbio.03944-25-s0003.pdf Figure S9.

File S4 mbio.03944-25-s0004.pdf Figure S10.

File S5 mbio.03944-25-s0005.pdf Figures S11 to S15.

File S6 mbio.03944-25-s0006.pdf Figures S16 to S18, Table S1, legend for Movie S1, and supplemental references.

Movie S1 mbio.03944-25-s0007.mp4 Time-lapse imaging of colony growth based on optical density measurements.

ASM does not own the copyrights to Supplemental Material that may be linked to, or accessed through, an article. The authors have granted ASM a non-exclusive, world-wide license to publish the Supplemental Material files. Please contact the corresponding author directly for reuse.
