# Supplementary material for: Class E sortase SrtE and two SrtE-dependent cell wall-anchored hydrophobic proteins are involved in morphogenesis in Actinoplanes missouriensis: occurrence of exploratory growth beyond genus Streptomyces
Source: mBio. 2026 May 18;17(6):e03944-25. doi: 10.1128/mbio.03944-25 (PMC13251363; doi:10.1128/mbio.03944-25)
Supplement: File S1 — Supplemental material title page; Figures S1 to S5. [file mbio.03944-25-s0001.pdf]

Supplemental material for

Class E sortase SrtE and two SrtE-dependent cell wall-anchored hydrophobic proteins are involved in morphogenesis in *Actinoplanes missouriensis*: occurrence of exploratory growth beyond genus *Streptomyces*

Zhuwen Tan,[1] Kazuki Nosho,[1,2] Kyohei Umebayashi,[3] Kenji Akamatsu,[3] Reiichi Ariizumi,[3,#] Takeaki Tezuka,[1,2] Yasuo Ohnishi[1,2]

[1]Department of Biotechnology, Graduate School of Agricultural and Life Sciences, The University of Tokyo, Bunkyo-ku, Tokyo, Japan

[2]Collaborative Research Institute for Innovative Microbiology, The University of Tokyo, Bunkyo-ku, Tokyo, Japan

[3]CarbGeM Inc., Shibuya-ku, Tokyo, Japan

[#]Present address: EXORPHIA Inc., Chiyoda-ku, Tokyo, Japan

This PDF file includes:

Figures S1 to S18
Table S1
Legend for Movie S1
Supplemental references

Other supporting material for this manuscript includes the following:

Movie S1

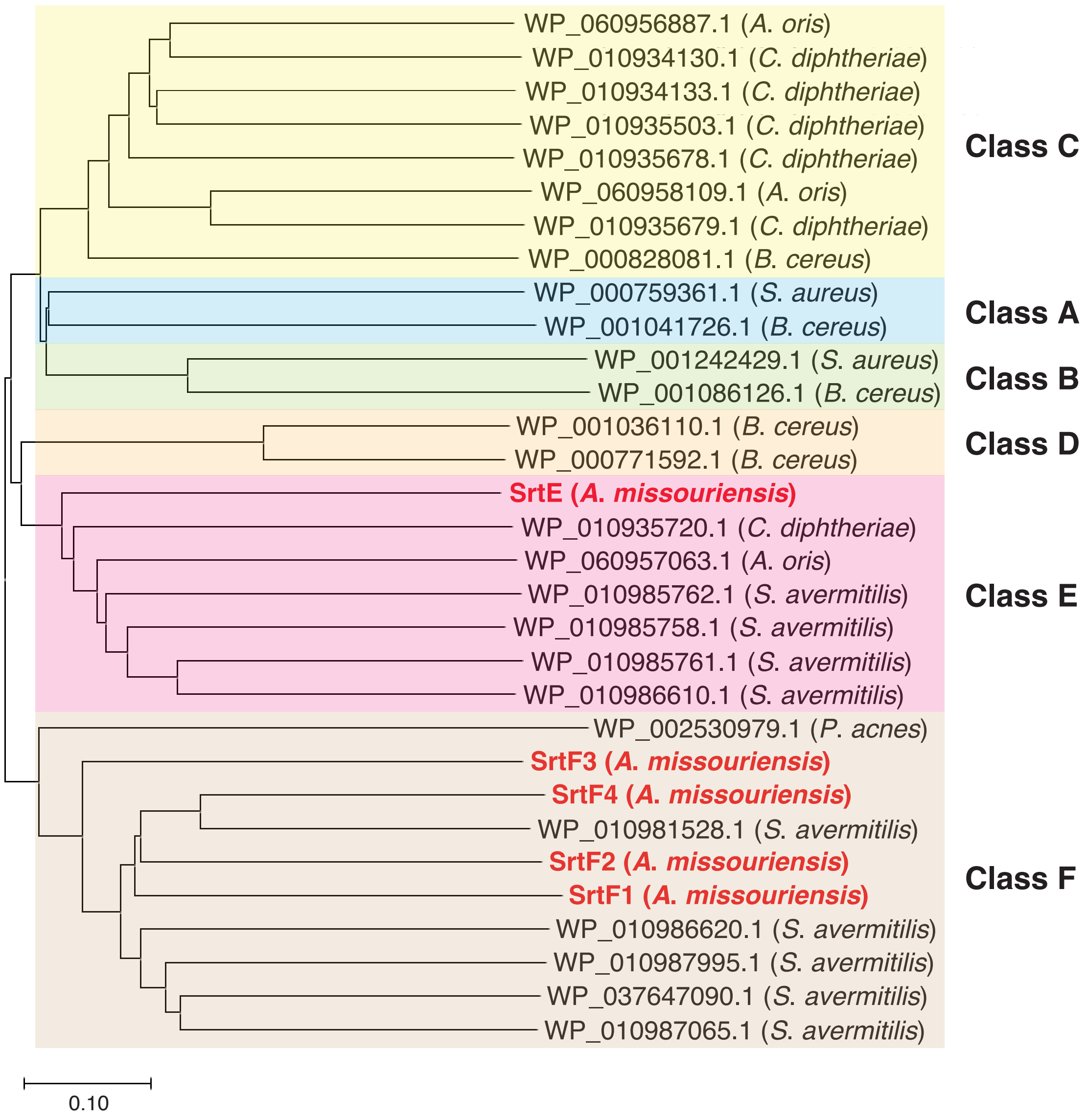

Fig. S1. Phylogenetic relationships among sortases. A neighbor-joining tree was constructed using MEGA X (Kumar *et al*., 2018). Three proteins from *Actinomyces oris*, two proteins from *Staphylococcus aureus*, five proteins from *Bacillus cereus*, six proteins from *Corynebacterium diphtheriae*, one protein from *Propionibacterium acnes*, nine proteins from *Streptomyces avermitilis*, and five proteins from *Actinoplanes missouriensis* (31 proteins in total) were used. Proteins from *A. missouriensis* are indicated in red letters. Members of classes A–F are shown with different colored backgrounds.

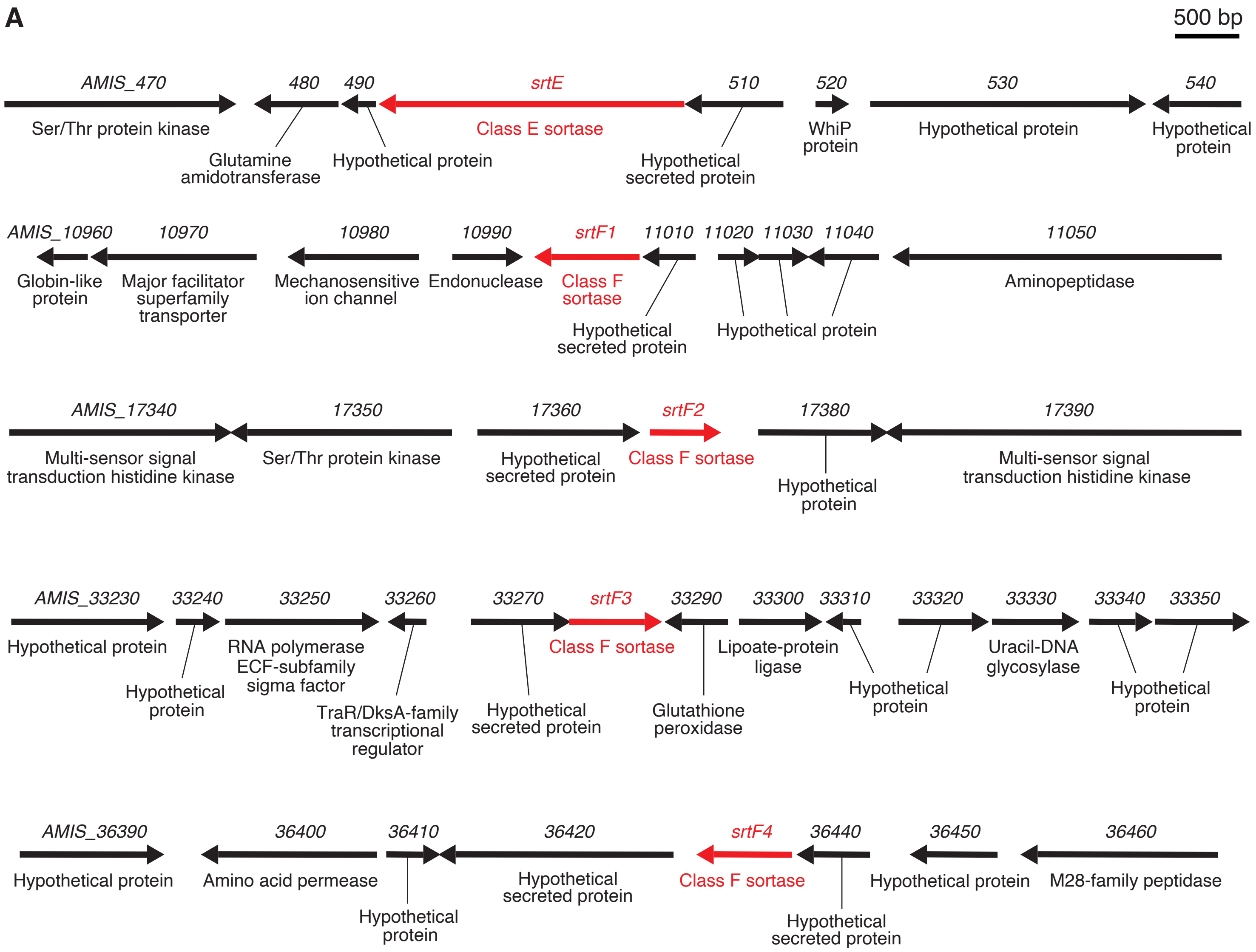
A
500 bp
AMIS_470
Ser/Thr protein kinase
480
Glutamine amidotransferase
490
Hypothetical protein
srtE
Class E sortase
510
Hypothetical secreted protein
520
WhiP protein
530
Hypothetical protein
540
Hypothetical protein
AMIS_10960
Globin-like protein
10970
Major facilitator superfamily transporter
10980
Mechanosensitive ion channel
10990
Endonuclease
srtF1
Class F sortase
11010
Hypothetical secreted protein
11020
11030
11040
Hypothetical protein
11050
Aminopeptidase
AMIS_17340
Multi-sensor signal transduction histidine kinase
17350
Ser/Thr protein kinase
17360
Hypothetical secreted protein
srtF2
Class F sortase
17380
Hypothetical protein
17390
Multi-sensor signal transduction histidine kinase
AMIS_33230
Hypothetical protein
33240
Hypothetical protein
33250
RNA polymerase ECF-subfamily sigma factor
33260
TraR/DksA-family transcriptional regulator
33270
Hypothetical secreted protein
srtF3
Class F sortase
33290
Glutathione peroxidase
33300
Lipoate-protein ligase
33310
Hypothetical protein
33320
33330
Uracil-DNA glycosylase
33340
33350
Hypothetical protein
AMIS_36390
Hypothetical protein
36400
Amino acid permease
36410
Hypothetical protein
36420
Hypothetical secreted protein
srtF4
Class F sortase
36440
Hypothetical secreted protein
36450
Hypothetical protein
36460
M28-family peptidase

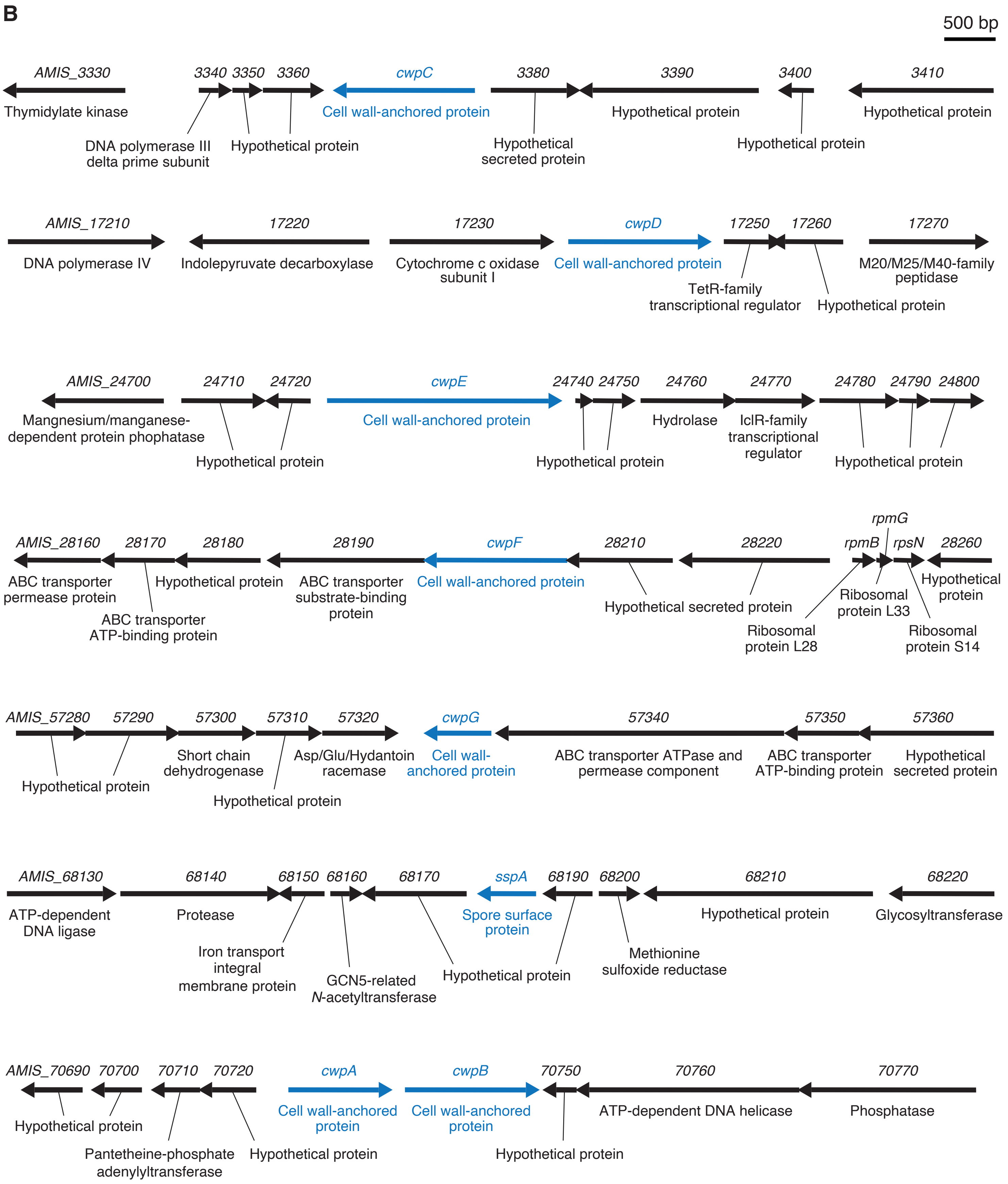

Fig. S2. Gene organization of five sortase genes (A), seven sortase substrate genes, and *sspA* (B) on the *A. missouriensis* genome. Arrows indicate the locations of the open reading frames, including their lengths and directions. Gene identification numbers or names are shown above the arrows. The putative functions of gene products are shown below the arrows. In (A), genes encoding sortases are colored red. In (B), genes encoding sortase substrates, including SspA, are colored light blue.

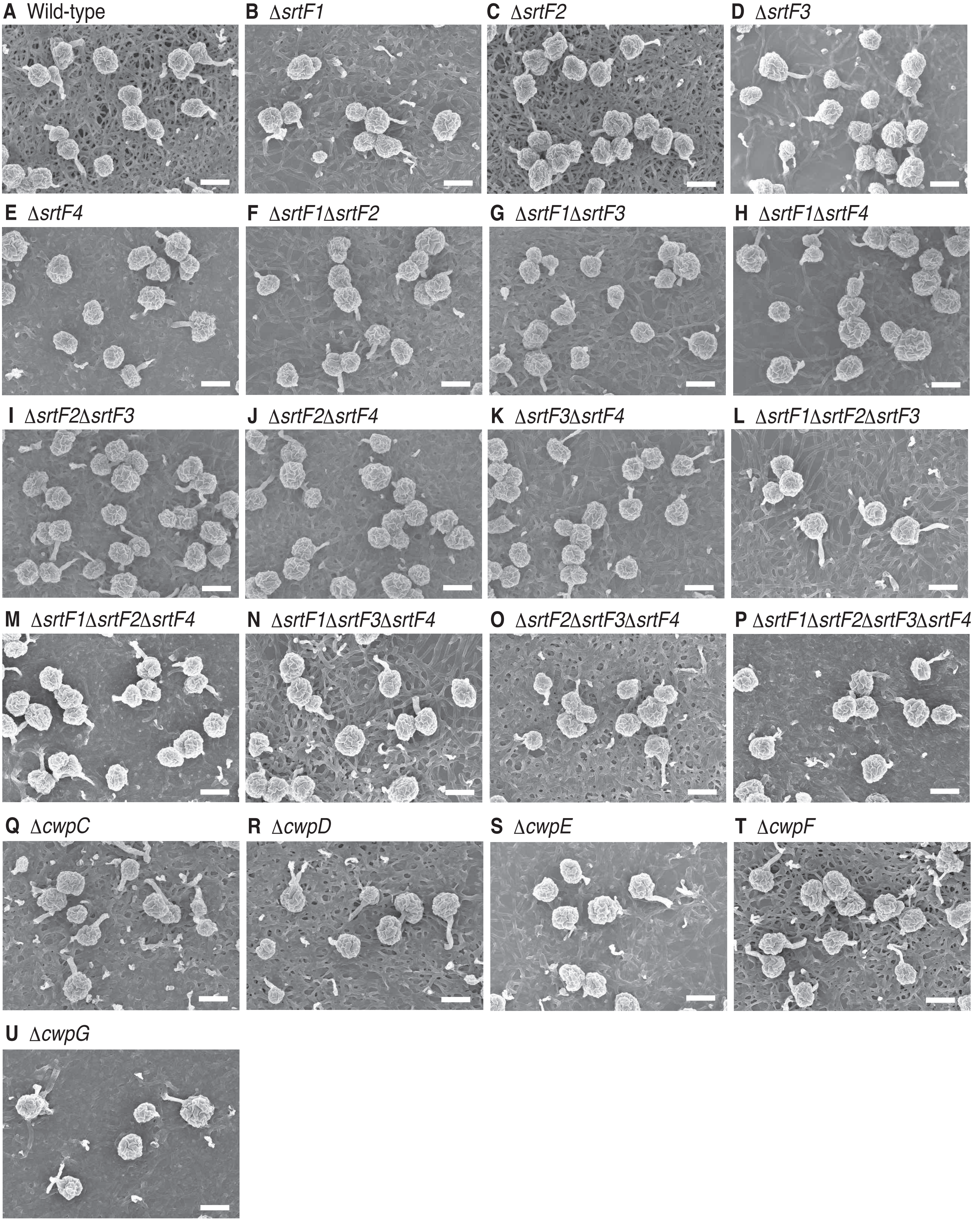

Fig. S3. SEM analysis of the wild-type and various mutant sporangia produced on HAT agar through 7 days of cultivation. Micrographs of the wild-type (A), Δ*srtF1* (B), Δ*srtF2* (C), Δ*srtF3* (D), Δ*srtF4* (E), Δ*srtF1*Δ*srtF2* (F), Δ*srtF1*Δ*srtF3* (G), Δ*srtF1*Δ*srtF4* (H), Δ*srtF2*Δ*srtF3* (I), Δ*srtF2*Δ*srtF4* (J), Δ*srtF3*Δ*srtF4* (K), Δ*srtF1*Δ*srtF2*Δ*srtF3* (L), Δ*srtF1*Δ*srtF2*Δ*srtF4* (M), Δ*srtF1*Δ*srtF3*Δ*srtF4* (N), Δ*srtF2*Δ*srtF3*Δ*srtF4* (O), Δ*srtF1*Δ*srtF2*Δ*srtF3*Δ*srtF4* (P), Δ*cwpC* (Q), Δ*cwpD* (R), Δ*cwpE* (S), Δ*cwpF* (T), and Δ*cwpG* (U) strains are shown. Bars, 5 μm.

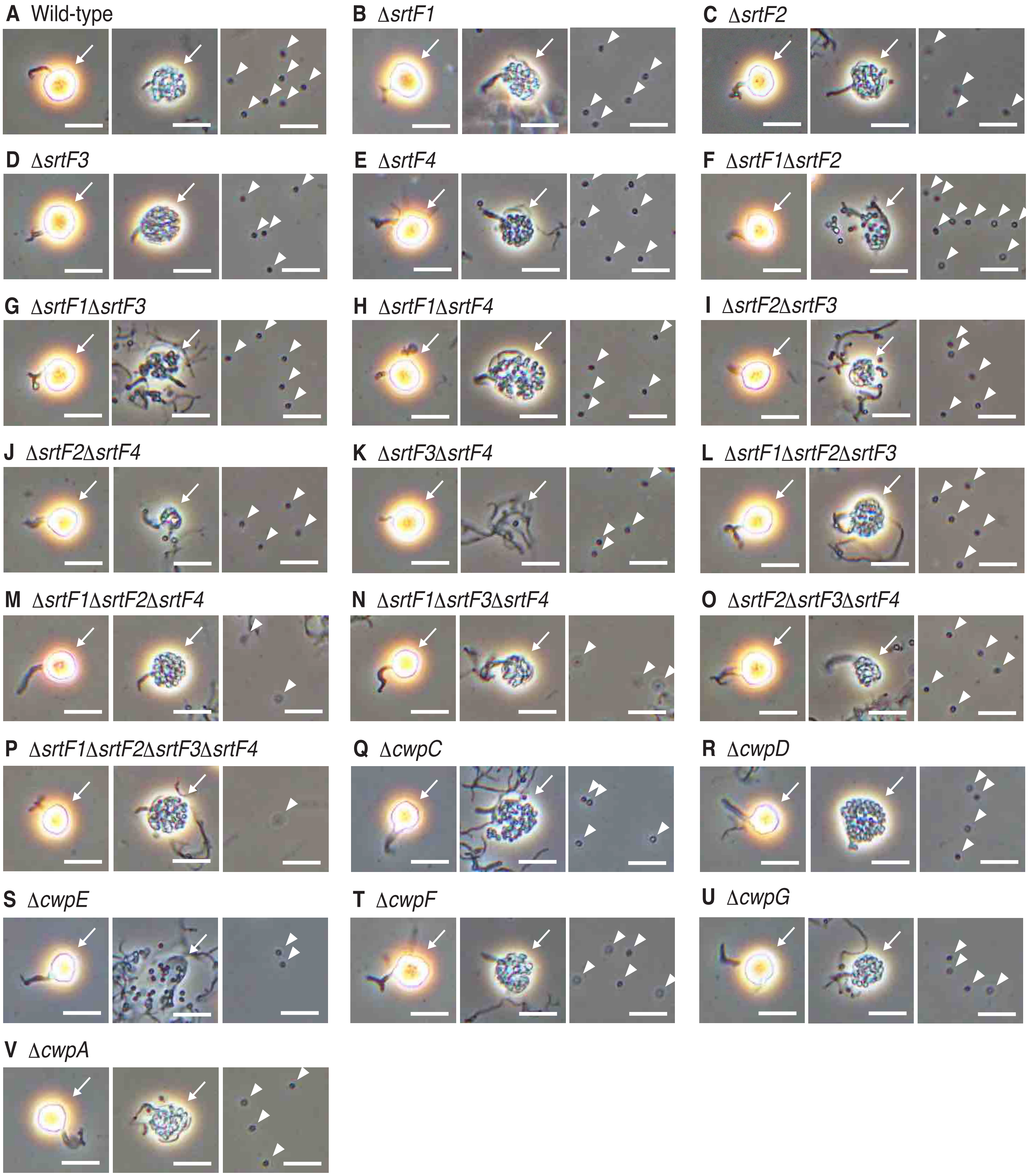

Fig. S4. Observation of sporangium dehiscence using phase-contrast microscopy. Sporangia of the wild-type and various mutant strains produced on HAT agar were harvested and suspended in 25 mM histidine solution to induce sporangium dehiscence. Micrographs of the wild-type (A), Δ*srtF1* (B), Δ*srtF2* (C), Δ*srtF3* (D), Δ*srtF4* (E), Δ*srtF1*Δ*srtF2* (F), Δ*srtF1*Δ*srtF3* (G), Δ*srtF1*Δ*srtF4* (H), Δ*srtF2*Δ*srtF3* (I), Δ*srtF2*Δ*srtF4* (J), Δ*srtF3*Δ*srtF4* (K), Δ*srtF1*Δ*srtF2*Δ*srtF3* (L), Δ*srtF1*Δ*srtF2*Δ*srtF4* (M), Δ*srtF1*Δ*srtF3*Δ*srtF4* (N), Δ*srtF2*Δ*srtF3*Δ*srtF4* (O), Δ*srtF1*Δ*srtF2*Δ*srtF3*Δ*srtF4* (P), Δ*cwpC* (Q), Δ*cwpD* (R), Δ*cwpE* (S), Δ*cwpF* (T), Δ*cwpG* (U), and Δ*cwpA* (V) strains are shown. Images in the left, middle, and right panels were obtained immediately, 20 min, and 60 min after the suspension, respectively. Sporangia (including those whose membrane became transparent) and released spores are indicated by arrows and arrowheads, respectively. Scale bars, 5 μm. The sporangia of all strains showed a normal sporangium dehiscence process, in which (i) sporangia appeared phase-bright immediately after suspension, (ii) the sporangium outer envelopes of many sporangia became transparent after 20 min of incubation, and (iii) sporangium dehiscence of many sporangia was completed after 60 min of incubation, with the release of zoospores.

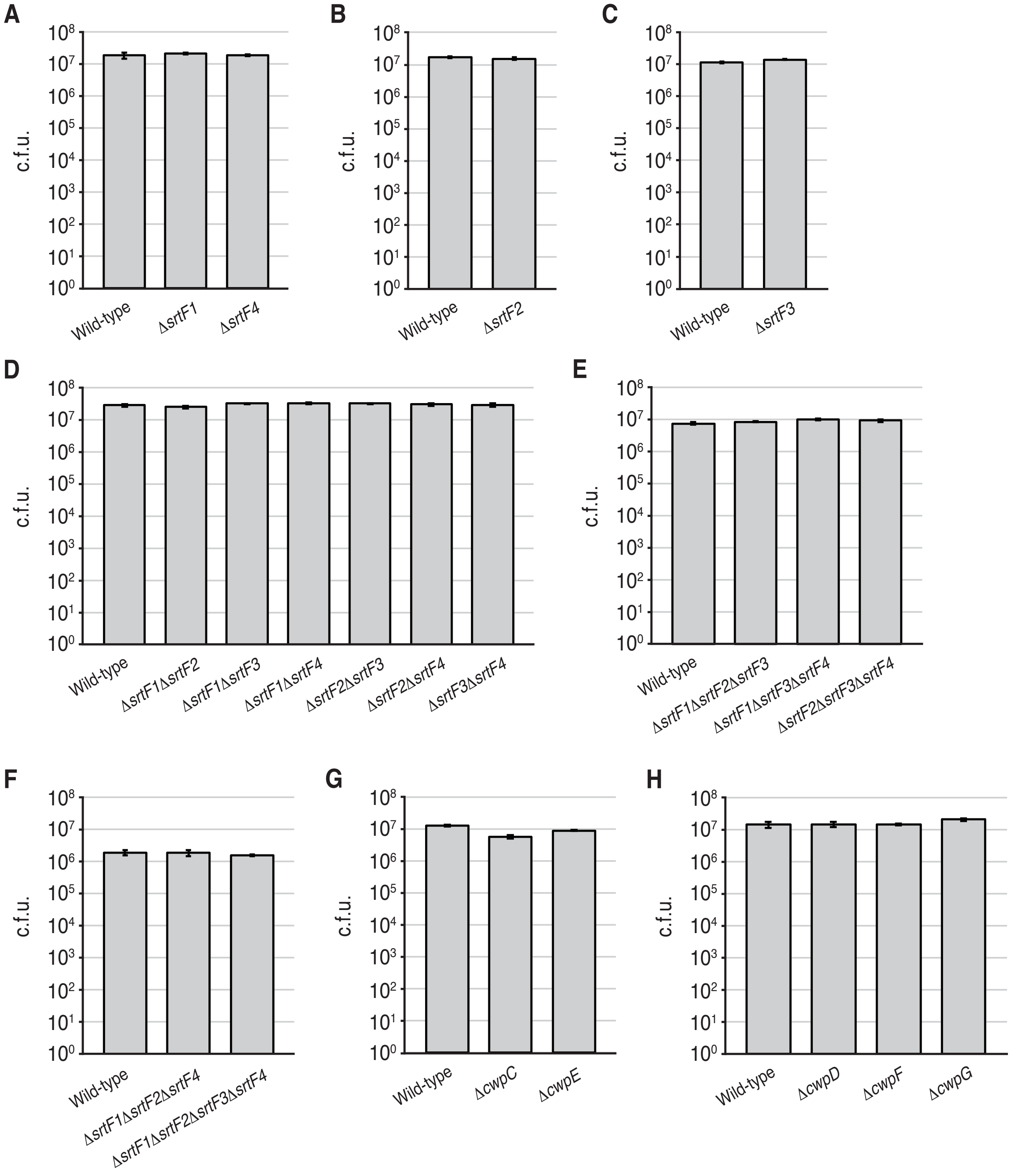

Fig. S5. Number of spores released from wild-type and various mutant sporangia. Each strain was cultivated on HAT agar at 30°C for 7 days. Zoospores released from sporangia by pouring 25 mM NH_4HCO_3 solution on the sporangium-forming agar were counted as colony-forming unit (c.f.u.) values on YBNM agar. The values represent mean ± standard error of three biological replicates. (A) Wild-type, Δ*srtF1*, and Δ*srtF4* strains. (B) Wild-type and Δ*srtF2* strains. (C) Wild-type and Δ*srtF3* strains. (D) Wild-type, Δ*srtF1*Δ*srtF2*, Δ*srtF1*Δ*srtF3*, Δ*srtF1*Δ*srtF4*, Δ*srtF2*Δ*srtF3*, Δ*srtF2*Δ*srtF4*, and Δ*srtF3*Δ*srtF4* strains. (E) Wild-type, Δ*srtF1*Δ*srtF2*Δ*srtF3*, Δ*srtF1*Δ*srtF3*Δ*srtF4*, and Δ*srtF2*Δ*srtF3*Δ*srtF4* strains. (F) Wild-type, Δ*srtF1*Δ*srtF2*Δ*srtF4*, and Δ*srtF1*Δ*srtF2*Δ*srtF3*Δ*srtF4* strains. (G) Wild-type, Δ*cwpC*, and Δ*cwpE* strains. (H) Wild-type, Δ*cwpD*, Δ*cwpF*, and Δ*cwpG* strains.
