# Supplementary material for: Class E sortase SrtE and two SrtE-dependent cell wall-anchored hydrophobic proteins are involved in morphogenesis in Actinoplanes missouriensis: occurrence of exploratory growth beyond genus Streptomyces
Source: mBio. 2026 May 18;17(6):e03944-25. doi: 10.1128/mbio.03944-25 (PMC13251363; doi:10.1128/mbio.03944-25)
Supplement: File S2 — Figures S6 to S8. [file mbio.03944-25-s0002.pdf]

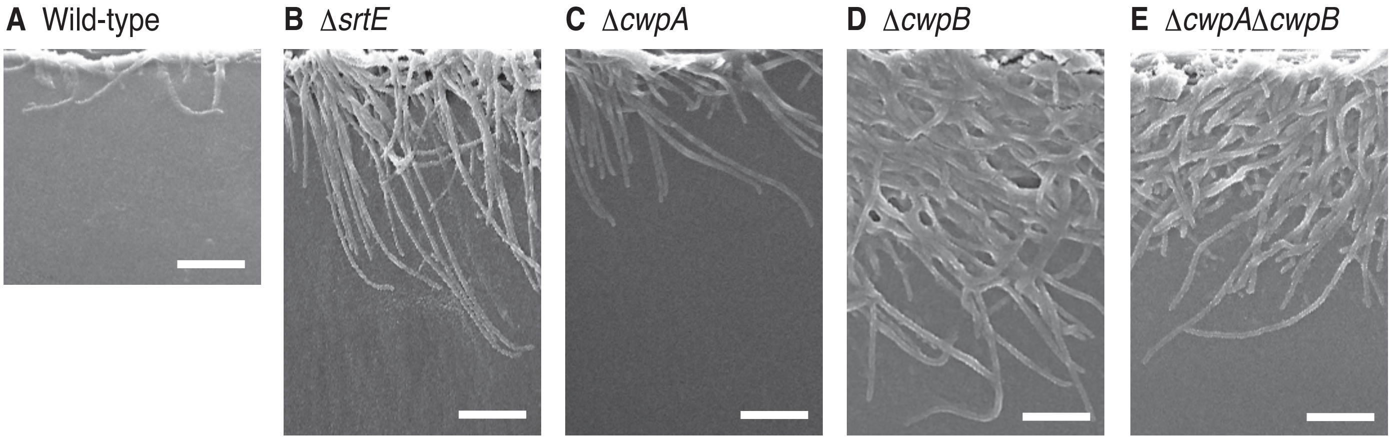

Fig. S6. SEM analysis of the edges of colonies grown on agar medium. The wild-type and mutant strains were cultivated on YBNM agar at 30°C for 11 days. (A) Wild-type strain. (B) Δ*srtE* strain. (C) Δ*cwpA* strain. (D) Δ*cwpB* strain. (E) Δ*cwpA*Δ*cwpB* strain. Bars, 10 μm.

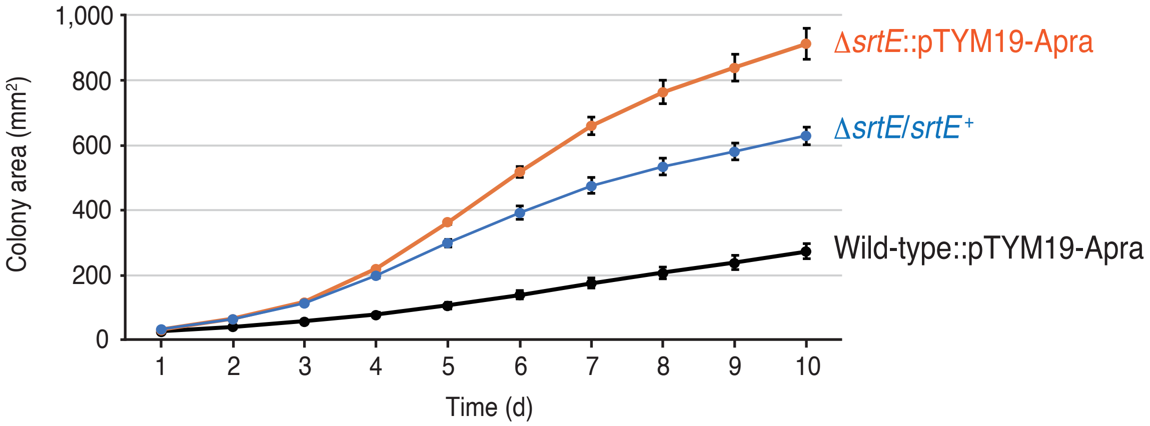

Fig. S7. Quantification of the areas of colonies formed on agar medium. The wild-type and Δ*srtE* strains, both of which harbored the empty vector pTYM19-Apra, and the Δ*srtE* strain harboring the *srtE* complementation plasmid were cultivated on YBNM agar at 30°C for 10 days. Colony areas were measured at 24 h intervals. The values represent mean ± standard error of three biological replicates.

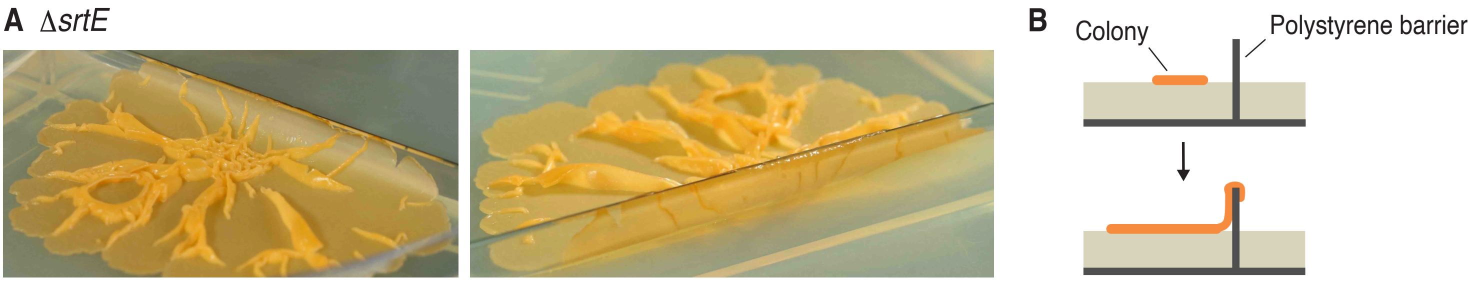

Fig. S8. Behavior of the Δ*srtE* colony on agar medium. (A) The Δ*srtE* colony growing over a polystyrene barrier. The Δ*srtE* cell suspension (10 μl) was inoculated on YBNM agar in a divided Petri dish and cultivated at 30°C for 10 days. (B) Schematic representation of Δ*srtE* colony growth during cultivation.
