# Supplementary material for: Class E sortase SrtE and two SrtE-dependent cell wall-anchored hydrophobic proteins are involved in morphogenesis in Actinoplanes missouriensis: occurrence of exploratory growth beyond genus Streptomyces
Source: mBio. 2026 May 18;17(6):e03944-25. doi: 10.1128/mbio.03944-25 (PMC13251363; doi:10.1128/mbio.03944-25)
Supplement: File S3 — Figure S9. [file mbio.03944-25-s0003.pdf]

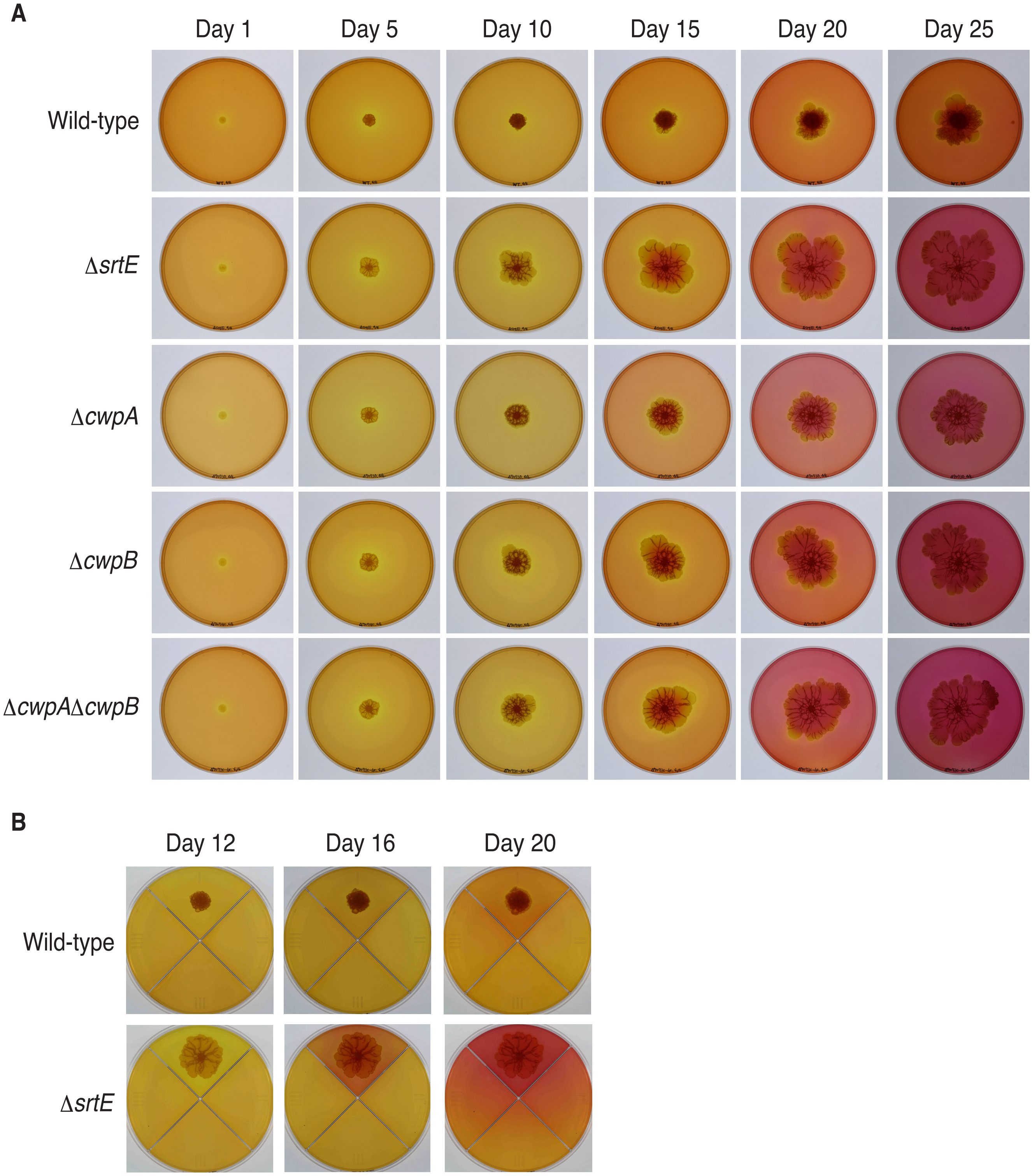

Fig. S9. Effects of colony growth on the pH of the agar medium. The wild-type and mutant strains were grown on YBNM agar containing 25 mg/l phenol red (pH indicator dye). The red color indicates the alkalinity of the medium. (A) Changes in pH during colony growth. The wild-type, Δ*srtE*, Δ*cwpA*, Δ*cwpB*, and Δ*cwpA*Δ*cwpB* strains were grown at 30°C for 25 days. Photographs were taken at 4- or 5-days intervals. (B) Effect of colony growth on the pH of the physically separated medium. The wild-type and Δ*srtE* strains were grown at 30°C for 20 days. Four compartments in each plate are separated by polystyrene barriers and the mycelia of each strain were inoculated in a compartment of the plate. Photographs of the left, middle, and right panels were obtained on days 12, 16, and 20 of cultivation, respectively.
