# Supplementary material for: Class E sortase SrtE and two SrtE-dependent cell wall-anchored hydrophobic proteins are involved in morphogenesis in Actinoplanes missouriensis: occurrence of exploratory growth beyond genus Streptomyces
Source: mBio. 2026 May 18;17(6):e03944-25. doi: 10.1128/mbio.03944-25 (PMC13251363; doi:10.1128/mbio.03944-25)
Supplement: File S4 — Figure S10. [file mbio.03944-25-s0004.pdf]

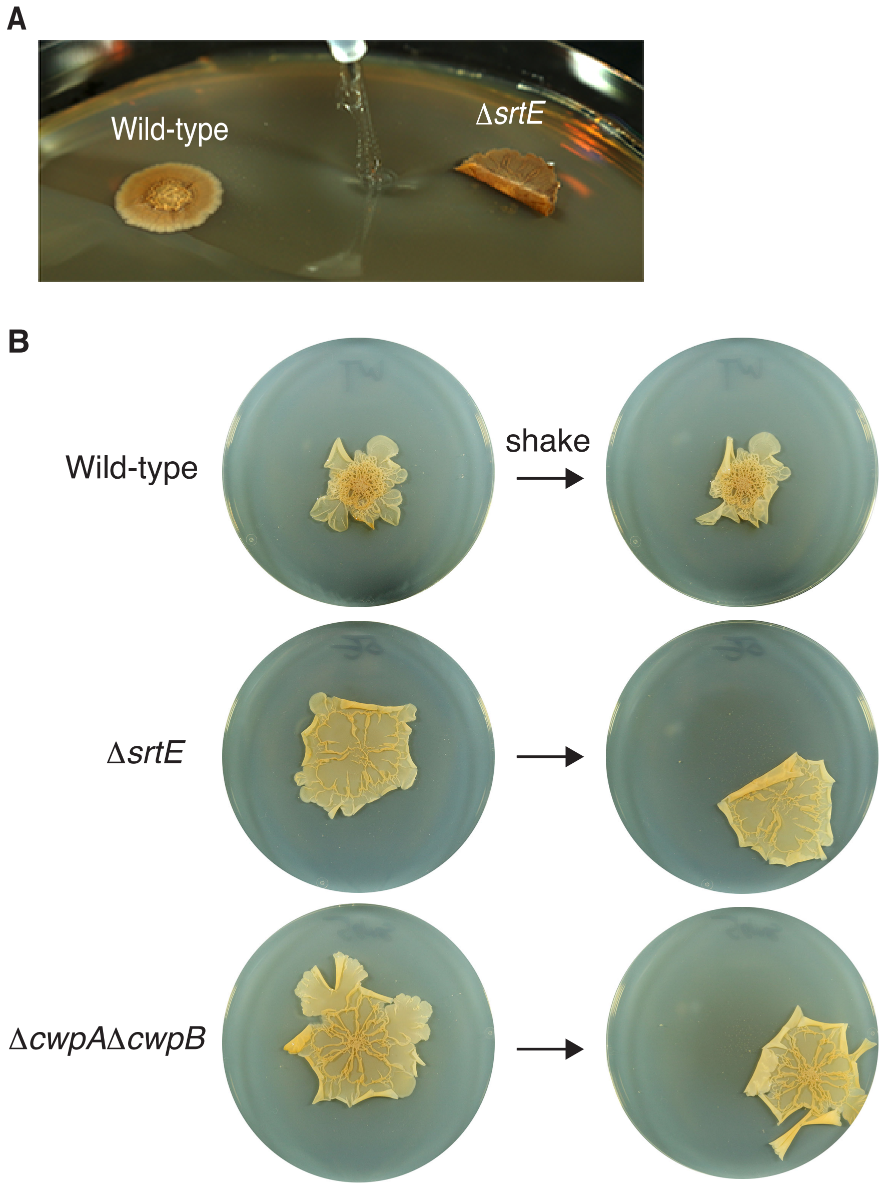

Fig. S10. Attachment of colonies to agar media. Wild-type and mutant strains were cultivated on HAT agar at 30°C for 10 days (A) and YBNM agar at 30°C for 20 days (B), and water was poured on each agar. (A) Wild-type and Δ*srtE* strains. The photograph was taken immediately after the onset of water pouring. (B) The wild-type, Δ*srtE*, and Δ*cwpA*Δ*cwpB* strains. Photographs of the left and right panels were taken immediately after pouring water and after subsequent gentle shaking of the plates, respectively.
