# Supplementary material for: Class E sortase SrtE and two SrtE-dependent cell wall-anchored hydrophobic proteins are involved in morphogenesis in Actinoplanes missouriensis: occurrence of exploratory growth beyond genus Streptomyces
Source: mBio. 2026 May 18;17(6):e03944-25. doi: 10.1128/mbio.03944-25 (PMC13251363; doi:10.1128/mbio.03944-25)
Supplement: File S5 — Figures S11 to S15. [file mbio.03944-25-s0005.pdf]

A CwpA

MNLPQSPLRRFGSLAAGTVLGLAGVAVVASPAFAHHSEVTGEYCKTASGDLNVTWNLDAVGQHVGEPRPYRLVTW
KPSPAGTVTNIAATEDENKFPYTAGTVVTGKQTITGAEPGTVPTVEYKAKFDNGHGDIDVRTAKAVDKGACAPTE
Disordered region
QPPAEEEPPPAEGEQPPASQSPSASPSASQSPSASPSASEEPEIPTEDIPVDADIKEIFEVTCDTMTIGLDNPAD
SLPINLHYKTSKGEERDLTINPGEAKSEKFSASEGFTVDLTISISAEGETFSETVTVPWEKPAGEDCDGGTGGGL
TM helix
PVTGAAAGGIAGGAAALLAVGAVLFMLARRRKVKFTA

B CwpB

MRTTPLRRAALVAAGALLGLTGLIGVASPASATNHGSEPLPGCVSADKAPLSVSGWQKGKTDAVVKYDGPAPLCQ
DLVVNMLTYTATSSSFKLPQYLFDSQTLQIKKDATSGTLEFSGAEVPGCFAQIDLVRGERIINPLKSDNDLYGDR
KLKWWNGGEGECSAKPVAEPIADCTGAASVKLINPQTTKETTFDVTAEGGFTKTVVIEPNDFGTVEIPADNAKNI
Disordered region
VVKANGKELYNGTPSKPEDCPKPQPTPAVLTPSSTCDGLTFTVKNPGDGKDVTVTFTPSQGNGDAKVVTLKPGQD
Disordered region
TEVTFPAKENLAVTVSGDITASNGVVTWTKPEECETPVKPSTPASEGPSEQPSEGPSEEPSATPSESVSESASPS
TM helix
VSATTSTTPVATTPVSDNEDGGSLPVTGAAAGGIAGGAALLLVVGAGLFFMARRRKLNFKA

C CwpC

MTPARSVAASVAALGFAGALTAPLPALAQNARCASPGSHAAQSSAQVLRINRLELGAVAKPATRAPEAGPPAVTG
Disordered region
VALGDVRSVLIAGGEVKSAAAARTLDGRVPGGSENDLVLQQAPPDNRAASRQRVGTKRFGPLRTGPGTLTAHARW
NNGLACASVTGEASESAAALNRVTLTGGGNQSLVRVPEAFSATSSTGLRRRGGEAESVSTTTLNAGKISLVDGEV
Disordered region
RIRVLRAATLRVSMSSGGRAEAAYEPAAVEITTRDGEQTLLDTPGDHHDVTLSEPSTPLESIPSPLASADPLPLP
SIPGVPAAPESENAPAPDSPTGAKLRISLGAARQATKGKAIAARATAIKILLVRGDDGPATTGHQSSAVVADLSL
Disordered region
TM helix
GVLDGAAVAPKRPTGTRAGTGSSNRTGTGTAAPAAAPGSPDVSPAGAAGLPITGPGLIPLLLAGSGMVIGGVCAF
LLSSRRRRES

D CwpD

MPIARRPIPLPRRRRAAETATAAVVAALVVPAVPAAAVTTDCRTAAFTARTQADLAKIAVLDPGPLHRDLPALAD
VRLGSSRGEADSGAKLHKAVATARYADAKLLGISTGQASAIATAPRSKGPASVDLATVGVAGLGVVKSGTATAEA
TWEDGYRCGKTGPLTRSATMLAGLSVLGGKHGAPVLHAVDDATHLSRKTSLLRAGPSGSTQSATDLVRIKGGQVG
VRAGAGAALGDLSLFAGTPHEIGIKVVNQATLEVVAAADQHKSRVDYKPAVLKVSSGGKQVNVLQDAGADLSLGV
DGSLSLPGGHHEKSGIGVRVSVGEATSDIGKRTVSAEAATVRVEVTVGKARLLDVALGYLSVSACAPGGGSSSDD
Disordered region
HGDDRDRGDHGPGSSPSSPGGSSSGDSSSGGSSSGGSSSGGSSSGGSSSEGPQGSAPADSASASPSGSASASPSV
TM helix
SVKPVALGEPLPANGGTGGGALALTGTNVAVLVIGGAALVLFGVGALMLGRRRRSVSGS

E CwpE

MSYLMRALAAAFCAVVLAFGSAPVAAAPAPPLPFTGQRCDSKFDLSGAPKKPDTIPVGDAGRLKANWDTYDFTNQ

RQSMDGLPAPTPQDLADAGDNPDIWKEGDKRKVYASYARRQGSSNPYGGTFKDWLNEVYIENAARNARGAAYRRK

VAFDFGMVGPHWLCEVQIKDANGKVIRTYDAVNTKTKEFIEFKSGGAHTGNQIPGDRAVMSDRRFAEYKLRYIFG

Disordered region

QEQEKKTTTALRNLGKAAGNTANGLPRVTAYEHRSTPIVRFTPGQYTKFDPTLNPNPGTGSGSRGGLNDMLNQSK

PTPETMRQQIERIRANDPTGQRLRGPGGVDFSTLELRFVGAPAKGKGLDYSFTAKKMPDPDENPGFGGEAKAQLI

SDSFFTWLALTPDKFWVNLNPDEPNRIMDAKFAKTDAGRVLLEADLQMKHDFAQAMNPEKPLGKKFWDATQLTDG

LPCLHSFRNWIEPAPAQVREQDGGIYILDAPLKVSSVPQNYTNLPPGSDRACKPTEAQIKHNHKAVLDVIVPEIE

RKINEDPYYADLRRVYTSRVAAEWIRQQDAKKATDYRAIINSNNVGKWPIRDGKWTTDDLWKRFLKSWNEGDYKF

EWKAGGEVYIYQMGGVDFSKSPKKNATKQQFTAKHQYLPRSTKVSVQTMTDDAESEEILLLGGNTDAKPTTGATK

Disordered region | TM helix

PPGGDGGDGGDGNGGDGNGGGDDDGGDDGDGSGGGATTPPADDTAGGLPITGDSVPVPVLVALAVTLIAAGAALV

WWARHRRRTFVS

F CwpF

MTRIAALATASLAVVLTAGGPALAAPPADGADLIALSVESGQLRMSFRGPGQAATDPQSVRLTTSGTPTGVIPDD

PAFGFLGRPGAPVWSLQEGSPFSTLDTTGIDEGTVALELADVDGPGSFAAYTLSPWGRPTLLLDSDDRTRATLPA

Disordered region

GQRLGGVVWLFDATGEYRVTVRASKRSGSRTLKDEALYTVSVPAAAGQKQTATRQKQAATQQKQATGEQKQTAKA

AAEQAPASAPKAAAAAGRKVISDGHVDMGPQLSGNKLTIRIKDDTTTPATWRELSDVTLKVTDKARIDVPAGAGY

ABC transporter-like

AFLGKAGEKVHLLPQSQQSGIVWPGWNTQHESVVKGTRGNVVWRLKQVDGPGEFKLFLTGSFGTPEVLFDSAKKL

Disordered region

PQQLGIAPNTHAHGNWAFTEAGVYKLTVEMTATTTAGTSVSDTKTLTLAVGDAANTGGGDTGGGNGAEPGPSASA

TM helix

GAGSGTGNGSGEGGGDGSGNGGGGGDLAMTGMNIVSIAGGGALLIAAGAATVALSRRRKENG

G CwpG

Neocarzinostatin-like

MLYRRLAAAAAAGLLCALSVGVTPAAAKATLKVSKTTGLKSGDSVTVSGTGFTKNLTDLALGQCVKNPKGPSDCN

LAGGAVFAKTDGSGKTDTVTLKLATSFSGKECGSDGCVIAAQLLPSSHDAATVAANAVSVKIVFGSSGGTAKPVT

TM helix

SKSSAAAATTTAAAADDPDSDSDSGSALPKTGPGMEWATVVLIGTGLLLPGAGVLAMLPARRRRMAGFR

H SspA

MERTFSDRTDTPLMSFSRRLTAGIPAVGVTVLAALALSGSPATAATDAAQAARPGAVESSAPAATRGADDYNGDT

Disordered region

GAEQPATAPPAAATATSDTTGGTRGKPGYGGESPTTAPPATTPPTTPPGTVQATPPAGVSSETASPAPAVTTQGA

TM helix

GVSSGSTLPVTGAPLAGTLALGGLLVAAGVGAVLYTRRRRA

I AMIS_11010

MRLSATRLALALSFALVPALVPAGTPARAAAAVFVELNPSTVPAGDEVSLRASCDDNLKPATVTAEPIGEVTVQP

TM helix

EFGFLTATVRVPSDTEPGDFPVALRCPDGGGNATATLHVVADVEPARGPATGGGGTAPGPAASILVGGGFAAIAA

GLVLAVVSLRRRRIG

J AMIS_36440

MSYSHAAPARRGRRDRAIALGVGLLLALLLLFIVLATCDNNGDNTGQPVPGASSSGPSGNPGPGASIDPGGGGPS

Disordered region

NGSPDPGGVDPYPSGNAPSNGGNNGGGNNGGGDNGGGNNGGENGGGNNGGDDDEGNGSGGDDGSATTAPTPKGGV

TM helix

DAGGGSGAEDRRVPFLVMGVFLLLAASGTAAYAVGRRPRA

Fig. S11. Amino acid sequences of putative sortase-dependent cell surface proteins. Predicted cleavage sites of signal peptides are indicated by arrowheads. Sortase-recognizing pentapeptide motifs are underlined. The locations of the transmembrane (TM) helices (all proteins), disordered regions (CwpA, CwpB, CwpC, CwpD, CwpF, SspA, and AMIS_36440), ABC transporter-like domain (CwpF), and neocarzinostatin-like domain (CwpG) are shown above the sequences. The positively charged residues in the C-terminal region are shown in red.

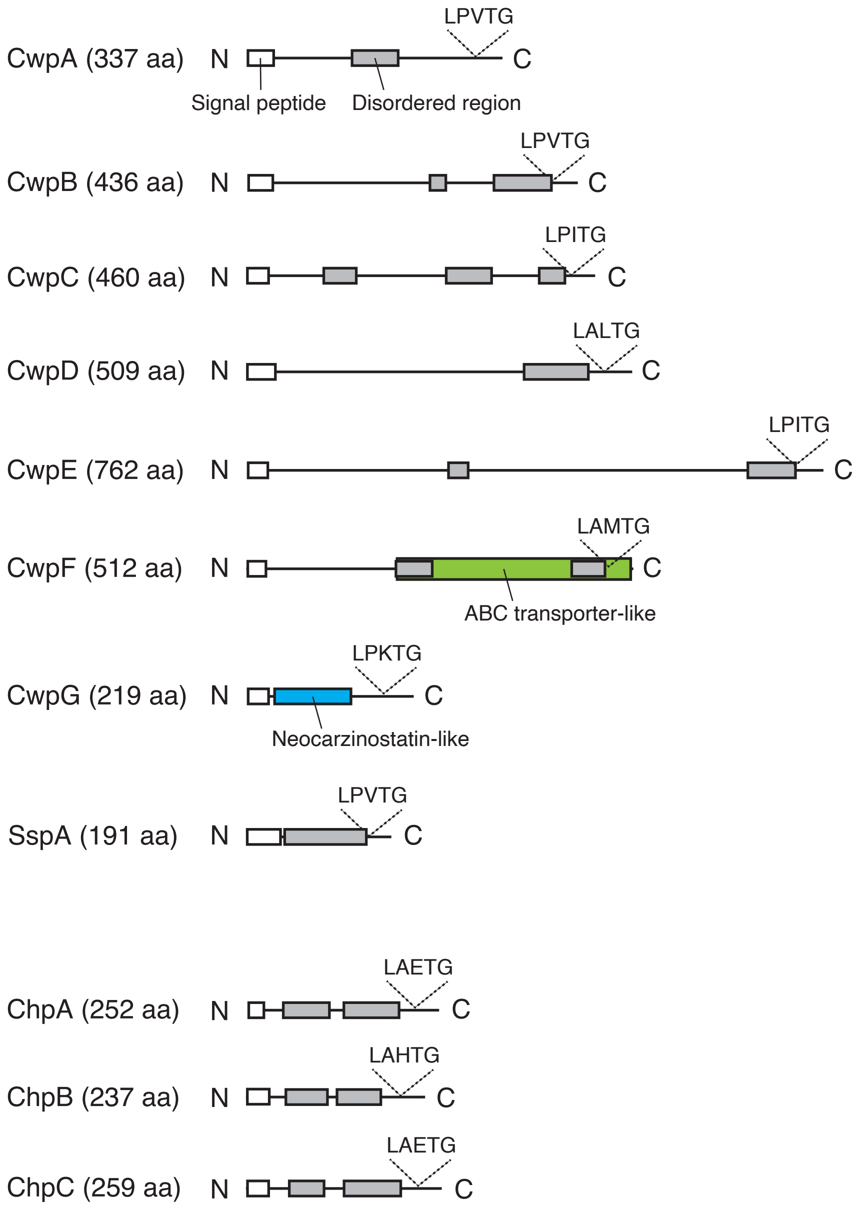

Fig. S12. Domain organization of full-length CwpA–G and SspA from *A. missouriensis* and ChpA, ChpB, and ChpC from *S. coelicolor* A3(2). The locations of the signal peptides, disordered regions, and sortase-recognizing pentapeptide motifs are indicated. In CwpF and CwpG, the location of the ABC transporter-like and neocarzinostatin-like domains, respectively, is also indicated.

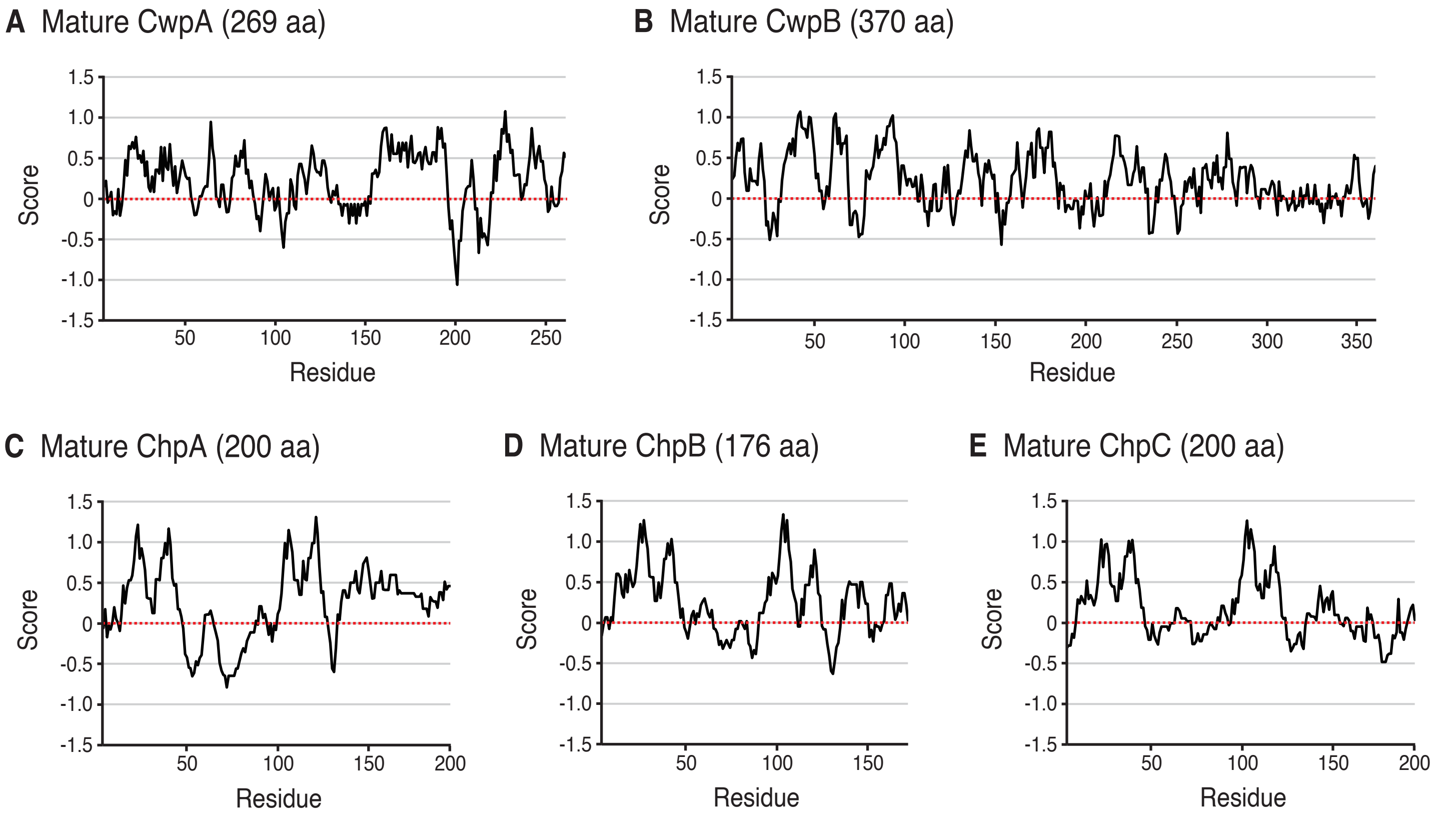

Fig. S13. Hydrophobic profiles of sortase-dependent surface proteins. The hydrophobicity scores of the mature polypeptides of CwpA and CwpB from *A. missouriensis* and ChpA, ChpB, and ChpC from *S. coelocolor* A3(2) were calculated according to the method described by Abraham and Leo (Abraham and Leo, 1987). The average scores of CwpA, CwpB, ChpA, ChpB, and ChpC were 0.22, 0.21, 0.24, 0.25, and 0.16, respectively. Higher scores correlate with higher protein hydrophobicity.

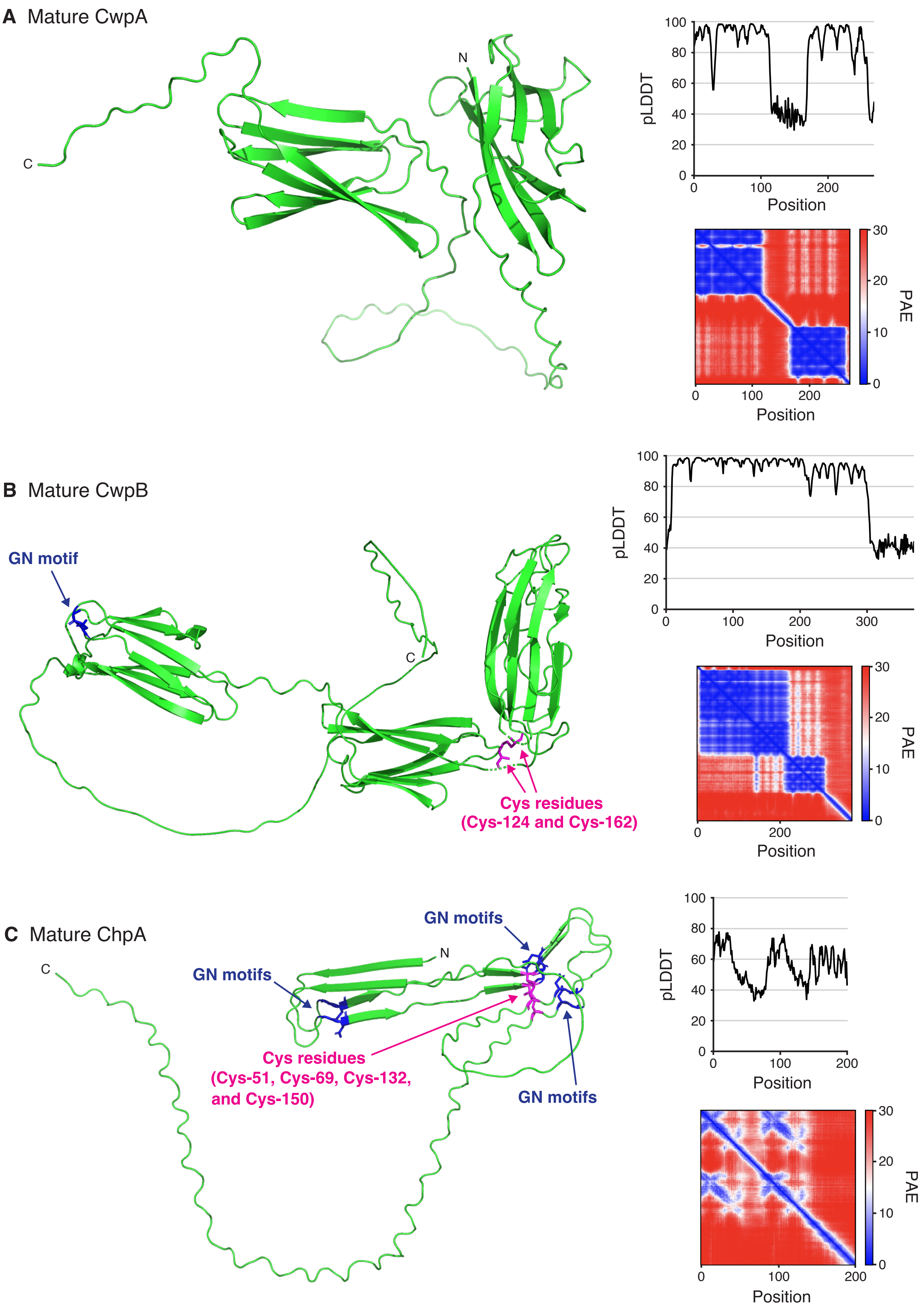

A Mature CwpA
C
N
pLDDT
Position
PAE
B Mature CwpB
GN motif
C
Cys residues
(Cys-124 and Cys-162)
C Mature ChpA
GN motifs
Cys residues
(Cys-51, Cys-69, Cys-132,
and Cys-150)

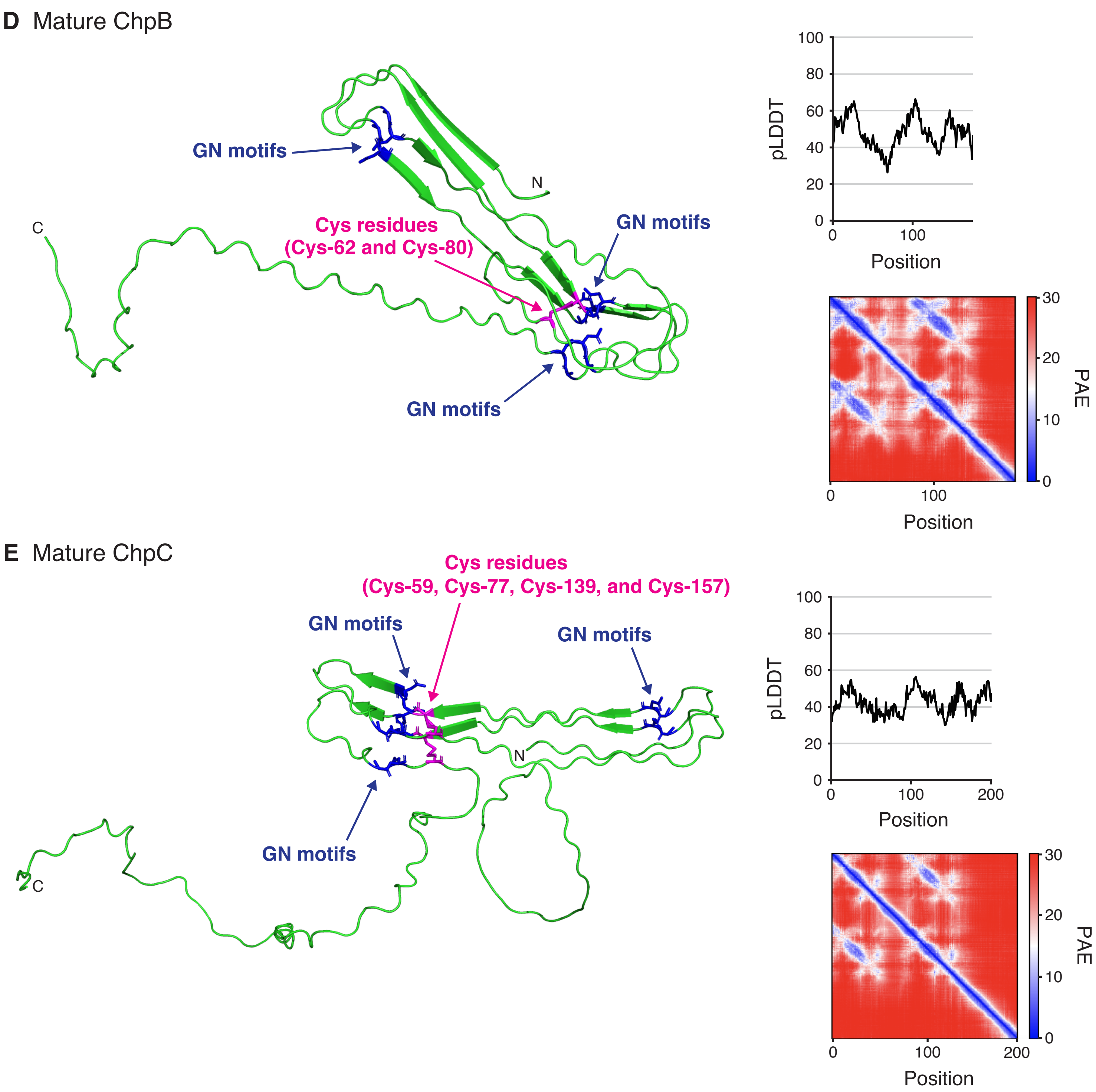

Fig. S14. AlphaFold-based prediction of the structures of CwpA (A) and CwpB (B) from *A. missouriensis* and ChpA (C), ChpB (D), and ChpC (E) from *S. coelicolor* A3(2). The sequences of mature proteins, in which the N-terminal signal sequences and the C-terminal sequences removed by the sortase were absent, were used for prediction (residues 35–303, 33–402, 21–220, 30–205, and 29–228 for CwpA, CwpB, ChpA, ChpB, and ChpC, respectively). Polypeptides are shown by ribbon representation and colored green. The predicted local distance difference test (pLDDT) score, which characterizes local structural accuracy, and the predicted aligned error (PAE) score, which corresponds to the topological accuracy between each residue, are shown on the right side of the predicted structures. In (B)–(E), the positions of the conserved GN motifs and cysteine residues are indicated in blue and magenta, respectively.

```
CwpA  --HHSEVTGEYCKTASGDLNVTWN---------------------------------------------LD
CwpB  TNHGSEPLPGCVSADKAPLSVSGWQKGKTDAVVKYDGPAPLCQDLVVNMLTYTATSSSFKLPQYLFDSQTL
ChpA  ------DSHADGAATNSPGAVSGN---------------------------------------------AL
ChpB  ----SDGAGAEGSAAGSPGLISGN---------------------------------------------TV
ChpC  ------DSGAHGTSSGSPGVLSGN---------------------------------------------TV
                            **

CwpA  AVGQHVGEPRPYRLVTWKPSPAGTVTNIAATEDENKFPYTAG-----------TVVTGKQTITGAEPGTVP
CwpB  QIKKDATSGTLEFSGAEVPGCFAQIDLVRGERIINPLKSDNDLYGDRKLKWWNGGEGECSAKPVAEPIADC
ChpA  QVPVDVP----------VNACGNTVDVIA---ALNPAFGN-----------------ECENASDEKTDGHG
ChpB  LPVDVP-----------VDVCGNTVNVVG---LLNPAAGN-----------------GCADSG--EPGASY
ChpC  QAPVHVP----------VNVCGNTVDVVG---VLNPAMGN-----------------ACANQGGGASGGHG
                          ***               **                  *
            Chaplin domain

CwpA  TVEYKAKFDNGHGDIDVRTAKAVDKGACAPTEQPPAE----------------------------------
CwpB  TGAASVKLINPQTTKETTFDVTAEGGFTKTVVIEPNDFGTVEIPADNAKNIVVKANGKELYNGTPSKPEDC
ChpA  GGYGEDASSSSSSSTSASSSGSHADGATE------------------------------------------
ChpB  QAAGASG-GTSGSATEATSGGAAAEGSGK------------------------------------------
ChpC  GHGGHGGY-GDSGGEGGSHGGSHAGGHAT------------------------------------------

CwpA  --EEPPPAEGEQPPASQSPSASPSASQSPSASPSASEEPEIPTEDIP----------VDADIKEIFEVTCD
CwpB  PKPQPTPAVL-TPSSTCDGLTFTVKNPGDGKDVTVTFTPSQGNGDAKVVTLKPGQDTEVTFPAKENLAVTV
ChpA  ----GSPGVG-SGNNAQVPVDVPVNLCGNTVDVIAALNPVFGN----------------------------
ChpB  ----DSPGVL-SGNGVQLPVHLPVNVSGNSVNVVGIGNPAVGN----------------------------
ChpC  ----DSPGVG-SGNHVEVPIDVPVNVCGNSIDVVGALNPTTGN----------------------------
                  **            ***            **
                                                       Chaplin domain

CwpA  TMTIGLDNPADSLPINLHYKTSKGEERDLTINPGEAKSEKFSASEGFTVDLTISISAEGETFSETVTVPWE
CwpB  SGDITASNGVVTWTKPEECETPVKPSTPASEGPSEQPSEGPSEEPSATPSESVSESASPSVSATTSTTPVA
ChpA  --KCENDAEEP---PGYGEEEPPPPTTPPGYGEEEPPPPTHEEPPPPSGEE---EPPPPSEEEHTPPAPQT
ChpB  --ESTNDSGD-----------------------HPEPVRPPAEPEPSAPEE---ERAGPGPSAHAAP-PRE
ChpC  --DCGNGGGGDHSTPPGDHETPPGEPHNPGNPGNPDTPDKPSGPDDETPGD---STDGNRPGAQTVDQPRG
         *

CwpA  KPAGEDCDGGTGGGLPVT
CwpB  TTPVSDNE--DGGSLPVT
ChpA  EQPPALAE--T-------
ChpB  EVSLAHT-----------
ChpC  DAALAET-----------
```

	Identity with CwpA	Similarity with CwpA	Identity with CwpB	Similarity with CwpB
CwpA	-	-	19%	29%
CwpB	19%	29%	-	-
ChpA	15%	20%	12%	18%
ChpB	12%	15%	14%	18%
ChpC	10%	13%	11%	15%

Fig. S15. Amino acid sequence alignment of sortase-dependent surface proteins. The mature polypeptides of CwpA and CwpB from *A. missouriensis* and ChpA, ChpB, and ChpC from *S. coelocolor* A3(2) are shown. Residues conserved in all polypeptides are indicated in white letters on black backgrounds. Two chaplin domains in ChpA, ChpB, and ChpC are shown below the alignment. The conserved GN motifs and Cys residues in the chaplin domain are indicated by blue and red asterisks, respectively, below the alignment. It should be noted that the Cys residues are not conserved in the second chaplin domain of ChpB. Four and eight Cys residues in CwpA and CwpB, respectively, are indicated in red letters. Sequence identities and similarities with CwpA or CwpB are shown at the end of the alignment.
