# Supplementary material for: Class E sortase SrtE and two SrtE-dependent cell wall-anchored hydrophobic proteins are involved in morphogenesis in Actinoplanes missouriensis: occurrence of exploratory growth beyond genus Streptomyces
Source: mBio. 2026 May 18;17(6):e03944-25. doi: 10.1128/mbio.03944-25 (PMC13251363; doi:10.1128/mbio.03944-25)
Supplement: File S6 — Figures S16 to S18, Table S1, legend for Movie S1, and supplemental references. [file mbio.03944-25-s0006.pdf]

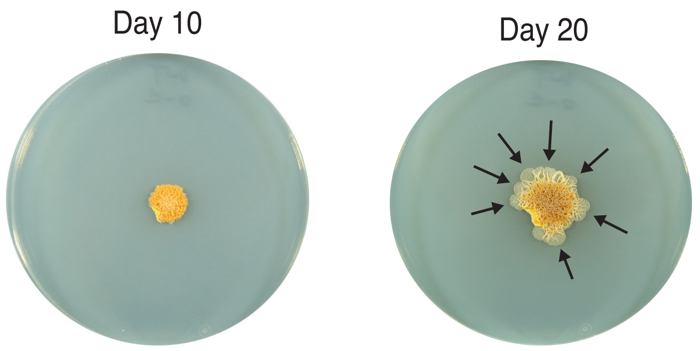

Fig. S16. Colony expansion on a nutrient-rich agar medium after prolonged incubation. The wild-type strain was cultivated on YBNM agar at 30°C for 20 days. Photographs of the left and right panels were obtained after 10 and 20 days of cultivation, respectively. In the right panel, arrows indicate the mycelia showing exploration.

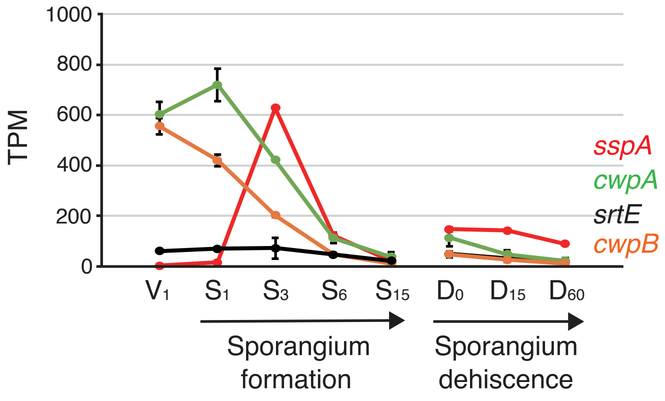

Fig. S17. Transcript levels of *srtE*, *cwpA*, *cwpB*, and *sspA*. Transcripts were examined using RNA-Seq analysis under various culture conditions. RNA samples were prepared from substrate hyphae grown on YBNM agar for 1 day (V_1), substrate hyphae or mixtures of substrate hyphae and sporangia grown on HAT agar for 1, 3, 6, and 15 days (S_1, S_3, S_6, and S_{15}, respectively), and sporangia (including some substrate hyphae) incubated in 25 mM histidine solution to induce sporangium dehiscence for 0, 15, and 60 min (D_0, D_{15}, and D_{60}, respectively). The average number of transcripts per million mapped reads (TPM) values ± standard error from three biological replicates are shown.

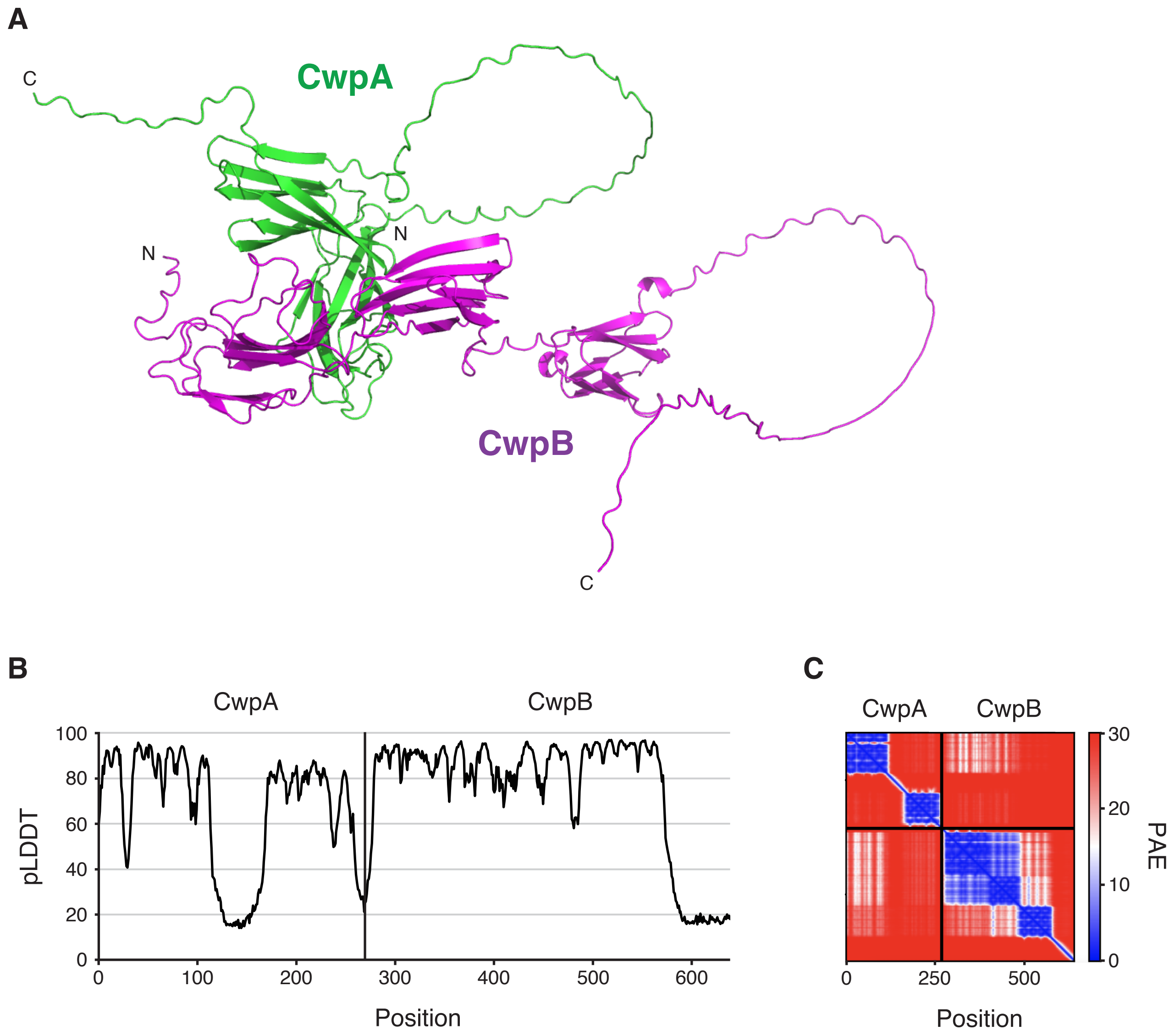

Fig. S18. AlphaFold-Multimer-based prediction of the CwpA-CwpB complex structure. (A) Predicted structure of the CwpA-CwpB heterodimer complex. The sequences of mature proteins, in which the N-terminal signal sequences and the C-terminal sequences removed by the sortase were absent, were used for prediction. Polypeptides are shown by ribbon representation and are colored green and magenta for CwpA and CwpB, respectively. (B) Predicted local distance difference test (pLDDT) score. (C) Predicted aligned error (PAE) score.

Table S1. Primers used in this study

Primer name	Sequence (5' to 3')[a]	Restriction enzyme	Used for
AMIS_500-UF1	GGAATTCCTCCACTTGAGCATCCTGGT	*Eco* RI	Disruption of *srtE*
AMIS_500-UR1	GCTCTAGAGTCGGACACGCGTGGGATGTA	*Xba* I	Disruption of *srtE*
AMIS_500-DF1	GCTCTAGATGACCGATGTACGCCTGGAT	*Xba* I	Disruption of *srtE*
AMIS_500-DR1	GCCAAGCTTGTCAGCTACCAGCTGTGCAA	*Hin* dIII	Disruption of *srtE*
AMIS_11000-UF1	GGAATTCCAGCTGATCGATCCCGATCA	*Eco* RI	Disruption of *srtF1*
AMIS_11000-UR1	GCTCTAGACACCCGGTCCTGACTCACCA	*Xba* I	Disruption of *srtF1*
AMIS_11000-DF2	GCTCTAGAGCCGACAACATCGTCGTCTT	*Xba* I	Disruption of *srtF1*
AMIS_11000-DR1	GCCAAGCTTCACGAAGCCGATCGGAATGT	*Hin* dIII	Disruption of *srtF1*
AMIS_17370-UF1	GGAATTCGGAAGTGTGCACTGCTTGAT	*Eco* RI	Disruption of *srtF2*
AMIS_17370-UR1	GCTCTAGAATCACCAGCACACTGAGGTA	*Xba* I	Disruption of *srtF2*
AMIS_17370-DF1	GCTCTAGAGTCATCGTCTATGCCGAGCT	*Xba* I	Disruption of *srtF2*
AMIS_17370-DR1	GCCAAGCTTCGAGATGATCGCCATGGTCA	*Hin* dIII	Disruption of *srtF2*
AMIS_33280-UF1	GGAATTCGCCCTCTACGACGTGCTGAT	*Eco* RI	Disruption of *srtF3*
AMIS_33280-UR2	GCTCTAGAGTCACCAGGGGTGATGCTCA	*Xba* I	Disruption of *srtF3*
AMIS_33280-DF1	GCTCTAGAACAACGTGGTGATCACCGCT	*Xba* I	Disruption of *srtF3*
AMIS_33280-DR1	GCCAAGCTTACTCACCATGACGACTTACT	*Hin* dIII	Disruption of *srtF3*
AMIS_36430-UF1	GGAATTCTACTCCATGCAGCCGATCGA	*Eco* RI	Disruption of *srtF4*
AMIS_36430-UR1	GCTCTAGACGGTCACGAATCTGCGGCAT	*Xba* I	Disruption of *srtF4*
AMIS_36430-DF1	GCTCTAGAGGTAGTTATCGGGACAACGT	*Xba* I	Disruption of *srtF4*
AMIS_36430-DR1	GCCAAGCTTTCGTTCTGGTAGAGCACGGT	*Hin* dIII	Disruption of *srtF4*
AMIS_3380-UF1	GGAATTCATCATCCAGACGCTGACCAT	*Eco* RI	Disruption of *cwpC*
AMIS_3380-UR1	GCTCTAGAACTGGCCAACGAAGGTCGAT	*Xba* I	Disruption of *cwpC*
AMIS_3380-DF1	GCTCTAGAGTCCACCTGTCAGGTCTGCA	*Xba* I	Disruption of *cwpC*
AMIS_3380-DR1	GCCAAGCTTCAAGCCGATCTTGGAGAGCT	*Hin* dIII	Disruption of *cwpC*
AMIS_17240-UF1	GGAATTCGATCATGTACCTGGTCACGT	*Eco* RI	Disruption of *cwpD*
AMIS_17240-UR1	GCTCTAGAGAAGATGTCCGATCTCTCAT	*Xba* I	Disruption of *cwpD*
AMIS_17240-DF2	GCTCTAGACGTTCGGCCCATGATCACTA	*Xba* I	Disruption of *cwpD*
AMIS_17240-DR1	GCCAAGCTTCGTGGAGGCTGTGACTGAGA	*Hin* dIII	Disruption of *cwpD*
AMIS_24730-UF1	GCTCTAGAGGAGCAGTCATTCGACGGAT	*Xba* I	Disruption of *cwpE*
AMIS_24730-UR1	AACTGCAGCAGGTCGAACTTGGAGTCGCA	*Pst* I	Disruption of *cwpE*
AMIS_24730-DF1	AACTGCAGACCTTCGTCAGCTGACCCGT	*Pst* I	Disruption of *cwpE*
AMIS_24730-DR1	GCCAAGCTTTATTCTGTTGGTCAGTGTGA	*Hin* dIII	Disruption of *cwpE*
AMIS_28200-UF1	GGAATTCACGAGAGATCCTCATGCGTA	*Eco* RI	Disruption of *cwpF*
AMIS_28200-UR1	GCTCTAGATGGTGTCGAGGGTGGAGAA	*Xba* I	Disruption of *cwpF*
AMIS_28200-DF1	GCTCTAGAGCATGAACATCGTCTCGAT	*Xba* I	Disruption of *cwpF*
AMIS_28200-DR1	GCCAAGCTTTGGACCTGGATCGTCCACTT	*Hin* dIII	Disruption of *cwpF*
AMIS_57330-UF1	GCTCTAGAAACATGCTCACCGGTGTCCT	*Xba* I	Disruption of *cwpG*
AMIS_57330-UR1	AACTGCAGTACCTCCAGAGTGAGTGTCA	*Pst* I	Disruption of *cwpG*
AMIS_57330-DF1	AACTGCAGATCGGAACCGGTCTGCTGCT	*Pst* I	Disruption of *cwpG*
AMIS_57330-DR1	GCCAAGCTTCTCCTGATGGACTATCCGAT	*Hin* dIII	Disruption of *cwpG*
AMIS_70730-40-UF1	GGAATTCAGCCGATCAGTTCTGCTTCA	*Eco* RI	Disruption of *cwpA*
AMIS_70730-40-UR1	GCTCTAGAATCTCCTGTGTAAGGGGTCA	*Xba* I	Disruption of *cwpA*
AMIS_70730-DF1	GCTCTAGAGGAGAACAACTTGCGAACCA	*Xba* I	Disruption of *cwpA*
AMIS_70730-DR1	GCCAAGCTTCTTGGAGAAGGGATGATCCT	*Hin* dIII	Disruption of *cwpA*
AMIS_70740-UF1	GGAATTCCACCACGTTGTCACGGATCA	*Eco* RI	Disruption of *cwpB*
AMIS_70740-UR1	GCTCTAGACAAGTGTGCGGTGGTACTTA	*Xba* I	Disruption of *cwpB*
AMIS_70730-40-DF1	GCTCTAGAGTACGGACTGACGGTCATCA	*Xba* I	Disruption of *cwpB*
AMIS_70730-40-DR1	GCCAAGCTTTACAGCTGACGCTCGTGCAA	*Hin* dIII	Disruption of *cwpB*
AMIS_500-SF	GGAATTCGTGAAGATCGCCAGCGAGAA	*Eco* RI	Complementation of *srtE*
AMIS_500-SR	GCCAAGCTTGAAGACCACGTACCAGAGCA	*Hin* dIII	Complementation of *srtE*
AMIS_70730_40-SF	GGAATTCGTTCGGTGCATGGAAGATCT	*Eco* RI	Complementation of *cwpA*
AMIS_70730-SR	GCCAAGCTTGGAACGAGAGGAGTGACGAA	*Hin* dIII	Complementation of *cwpA*
AMIS_70740-soeSpR1	TTCGCAAGTTATCTCCTGTGTAAGGGGTCA		Complementation of *cwpB*
AMIS_70740-soeSgF1	CACAGGAGATAACTTGCGAACCACCCCCCT		Complementation of *cwpB*
AMIS_70740-soeSgR1	GCCAAGCTTCTCGTTCTTACGGTTTGTTA	*Hin* dIII	Complementation of *cwpB*
AMIS_70730_40-SR	GCCAAGCTTGTGATGACCGTCAGTCCGTA	*Hin* dIII	Complementation of *cwpAB*

[a] The recognition sequences for restriction enzymes are underlined.

Movie S1 (separate file). Time-lapse imaging of colony growth based on optical density measurements. Suspensions of the wild-type and Δ*srtE* mycelia were inoculated onto YBNM agar and incubated at 30°C for 10 days. The optical density was measured with a resolution of 100 × 100 μm at 5 min intervals. The data at 4 h intervals are visualized using heatmaps, and the color range is shown on the right side of the images.

Supplemental references

Kumar S, Stecher G, Li M, Knyaz C, Tamura K. 2018. MEGA X: Molecular evolutionary genetics analysis across computing platforms. Mol Biol Evol 35:1547-1549.

Abraham DJ, Leo AJ. 1987. Extension of the fragment method to calculate amino acid zwitterion and side chain partition coefficients. Proteins 2:130-152.
